# A novel PGPF *Penicillium olsonii* isolated from the rhizosphere of *Aeluropus littoralis* promotes plant growth, enhances salt stress tolerance, and reduces chemical fertilizers inputs in hydroponic system

**DOI:** 10.3389/fmicb.2022.996054

**Published:** 2022-10-27

**Authors:** Mohamed Tarroum, Walid Ben Romdhane, Fahad Al-Qurainy, Ahmed Abdelrahim Mohamed Ali, Abdullah Al-Doss, Lotfi Fki, Afif Hassairi

**Affiliations:** ^1^Department of Botany and Microbiology, College of Science, King Saud University, Riyadh, Saudi Arabia; ^2^Department of Plant Production, College of Food and Agricultural Science, King Saud University, Riyadh, Saudi Arabia; ^3^Laboratory of Plant Biotechnology Applied to Crop Improvement, Faculty of Sciences of Sfax, University of Sfax, Sfax, Tunisia; ^4^Centre of Biotechnology of Sfax, University of Sfax, Sfax, Tunisia

**Keywords:** *Penicillium olsonii*, halophilic PGPF, cell-free culture filtrate, salt stress, plant growth promotion, sustainable agriculture

## Abstract

The hydroponic farming significantly enhances the yield and enables multiple cropping per year. These advantages can be improved by using plant growth-promoting fungi (PGPF) either under normal or stress conditions. In this study, the fungal strain (A3) isolated from the rhizosphere of the halophyte plant *Aeluropus littoralis* was identified as *Penicillium olsonii* based on sequence homology of its ITS region. The A3 fungus was shown to be halotolerant (up to 1 M NaCl) and its optimal growth was at 27°C, but inhibited at 40°C. In liquid culture medium, the A3 produced indole acetic acid (IAA) especially in the presence of L-tryptophan. Tobacco plants grown under hydroponic farming system were used to evaluate the promoting activity of the direct effect of A3 mycelium (DE) and the indirect effect (IDE) of its cell-free culture filtrate (A3CFF). The results showed that for the two conditions (DE or IDE) the tobacco seedlings exhibited significant increase in their height, leaf area, dry weight, and total chlorophyll content. Interestingly, the A3CFF (added to the MS liquid medium or to nutrient solution (NS), prepared from commercial fertilizers) induced significantly the growth parameters, the proline concentration, the catalase (CAT) and the superoxide dismutase (SOD) activities of tobacco plants. The A3CFF maintained its activity even after extended storage at 4°C for 1 year. Since the A3 is a halotolerant fungus, we tested its ability to alleviate salt stress effects. Indeed, when added at 1:50 dilution factor to NS in the presence of 250 mM NaCl, the A3CFF enhanced the plant salt tolerance by increasing the levels of total chlorophyll, proline, CAT, and SOD activities. In addition, the treated plants accumulated less Na^+^ in their roots but more K^+^ in their leaves. The A3CFF was also found to induce the expression of five salt stress related genes (NtSOS1, NtNHX1, NtHKT1, NtSOD, and NtCAT1). Finally, we proved that the A3CFF can reduce by half the chemical fertilizers inputs. Indeed, the tobacco plants grown in a hydroponic system using 0.5xNS supplemented with A3CFF (1:50) exhibited significantly higher growth than those grown in 0.5xNS or 1xNS. In an attempt to explain this mechanism, the expression profile of some growth related genes (nitrogen metabolism (NR1, NRT1), auxin (TRYP1, YUCCA6-like), and brassinosteroid (DET2, DWF4) biosynthesis) was performed. The results showed that all these genes were up-regulated following plant treatment with A3CFF. In summary the results revealed that the halotolerant fungus *P. olsonii* can stimulates tobacco plant growth, enhances its salt tolerance, and reduces by half the required chemical fertilizer inputs in a hydroponic farming system.

## Introduction

Plants live in unstable environments that often inhibit growth and development. Unfavorable environmental conditions include biotic and abiotic stresses, such as drought, salt, and low or high temperatures, which greatly reduce plant productivity and threaten food security (Jamil et al., 2011; Zhu, 2016). Improving agricultural sustainability requires efficient management of soil fertility, fertilizer input, water use, and crop rotations, which help maintain proper soil equilibrium and high crop productivity. Biological processes in the soil play important roles in the cycling of essential elements for plant growth, including carbon, nitrogen, phosphorus, and sulfur (Drobek et al., 2019). In 2012, more than 6.2 million hectares in European countries were treated with biostimulants (Calvo et al., 2014; Colla and Rouphael, 2015). The use of microorganisms in improving nutrient availability for plants is an important strategy to protect biodiversity, and meet the increasing food demands of the global population (Vimal et al., 2017). It is well known that plant growth and health benefit from the presence of certain bacteria and fungi that live both within and around their tissues. In particular, soil fungi have long been known as a key element of soil fertility, plant nutrient turnover, and plant growth promotion (Haro and Benito, 2019). The plant growth-promoting fungi (PGPFs) can influence germination, seedling vigor, shoot growth, root growth, photosynthetic efficiency, flowering, and yield (López-Bucio et al., 2015; Hossain and Sultana, 2020b). Moreover, previous studies have revealed that PGPFs also improve systemic tolerance of abiotic stress in various crop plants (Khan et al., 2011; Zhang et al., 2016; Chandra and Singh, 2020). PGPFs include species of the genera *Aspergillus*, *Fusarium*, *Trichoderma*, *Penicillium*, *Piriformospora*, *Phoma*, and *Rhizoctonia* (Hossain et al., 2007, 2014, 2017; Shoresh et al., 2010). *Aspergillus*, *Fusarium*, *Penicillium*, *Piriformospora*, *Phoma*, and *Trichoderma* are specifically involved in abiotic stress tolerance (Chandra and Singh, 2020). The specific selection of halotolerant fungi that promote salt tolerance and plant growth could thus significantly enhance plant productivity either under normal or salt stress conditions, representing a promising approach for increasing nutrient bioavailability and helping advance saline soil-based agriculture.

The soil microorganisms play a fundamental role in regulating organic matter decomposition and the mobilization of nutrients for plants, thus promoting their growth and productivity (Yarzábal and Chica, 2017). PGPFs represent roughly 44.2% of rhizosphere fungal isolates (Hossain and Sultana, 2020a). However, their frequency of occurrence in the rhizosphere varies with host plant species (Schmid et al., 2018; Hakim et al., 2021). These beneficial fungi have a crucial role in physiological processes such as photosynthesis, phytohormone biosynthesis, and responses to salinity, drought, heat, cold and heavy metal stresses (Ansari et al., 2013). *Trichoderma virens* has been used to enhance biomass production and stimulate lateral root development in Arabidopsis seedlings (Contreras-Cornejo et al., 2009). A bushy root phenotype and strong stimulation of root hair development were observed in Chinese cabbage (*Brassica rapa*) seedlings treated with *Piriformospora indica* (Lee et al., 2011). Similarly, multiple species of PGPFs have been described that enhance cucumber (*Cucumis sativus*) plant growth *via* different mechanisms (Chandanie et al., 2009; Saldajeno and Hyakumachi, 2011; Hossain et al., 2014; Islam et al., 2014; Babu et al., 2015). Spinach (*Basella alba*) seedlings inoculated with the rhizosphere fungus *Fusarium* spp. showed more vegetative growth and both higher germination percentages and vigor indices compared to non-treated plants (Islam et al., 2014). Auxin and cytokinin phytohormones are involved in interactions between plant roots and soil microorganisms like bacteria and fungi (Boivin et al., 2016). The effectiveness of *Penicillium menonorum* on cucumber (*C. sativus*) growth is attributed to the secretion of indole acetic acid (IAA), which potentially represents the main mechanism for plant growth stimulation (Babu et al., 2015). Additionally, Arabidopsis seedlings showed characteristic auxin-related phenotypes, including lateral root development and biomass increases, when inoculated with *Trichoderma virens* (Contreras-Cornejo et al., 2009). Salas-Marina et al. (2011) also noted that the *Aspergillus ustus* promotes growth in *Arabidopsis thaliana* through auxin synthesis in liquid cultures.

Besides observing the direct effects on plant growth from mycelium, potential effects of fungal culture filtrates have also been studied. Treatments with *Fusarium tricinctum* RSF-4 l and *Alternaria alternata* RSF-6 l culture filtrates have been found to improve growth of rice (*Oryza sativa*) seedlings *via* IAA secreted into culture media (Khan et al., 2015). Autoclaved and filter-sterilized culture filtrates of *P. indica* enhance hairy root growth and lignin production of *Linum album* (Kumar et al., 2012). Furthermore, *in vitro* co-cultivation with *P. indica* culture filtrate alters callus production, organogenesis, and photosynthetic pigment content in *Artemisia* annua (Baishya et al., 2015). Khan et al. (2015) suggested that fungi secrete plant hormone-like compounds into culture media that help re-program and regulate endogenous phytohormone levels of the host plant. The *Penicillium* genus, which is widely distributed in soil habitats of cultivated, forest, desert, beach, and marine regions, has several species that are known PGPFs that can produce phytohormones and enhance mineral solubilization (Babu et al., 2015). Results from previous studies conducted with *Penicillium* sp. showed effective enhancement of plant growth and improvement of biotic and abiotic stress tolerance in multiple plant species (Hossain et al., 2007; Khan et al., 2008; Khan and Lee, 2013; Hossain et al., 2014; Radhakrishnan et al., 2014; Khan et al., 2015), and in one such study it was demonstrated that isolated *Penicillium* sp. cultures promote growth of rice seedlings and *Suada japonica* (You et al., 2012).

Hydroponics systems usually require lower nutrients and water use and, thus promote environmental protection and agricultural sustainability, especially when combined with plant growth promoting microorganisms (PGPMs) (Bartelme et al., 2018). In the current study, our main objective was to mine the rhizosphere of the halophyte plants for the presence of beneficial microorganisms that can help plants to grow under normal as well as under stress conditions. For this, a fungus (A3) belonging to the *Penicillium* genus was isolated from the rhizosphere of the halophyte plant *A. littoralis* as first step of this work. We secondly tested the effect of A3 mycelium on plant growth in either solid or liquid media. Next, the ability of A3 cell-free culture filtrate (A3CFF) to promote the growth and to alleviate salinity stress in tobacco plants grown in a hydroponic system was evaluated. We then determined the potential of A3CFF to decrease the quantity of chemical fertilizers required in nutrient solution (NS) in a hydroponic growing system. Finally, in order to understand the possible mechanisms by which A3CFF can promote plant growth and enhance salinity stress tolerance, the expression profiles of certain growth- and salt stress-related genes were monitored using semi-quantitative and quantitative RT-PCR.

## Materials and methods

### Fungi isolation and qualitative evaluation of growth-promoting activity

Following the method described by Tarroum et al. (2021), the fungi were isolated from the soil surrounding the roots of *A. littoralis*, plants that dominate the salty soil at a location called “Salboukh” (north of Riyadh, Saudi Arabia: 25°04′48.6” N 46°20′27.7″ E). Following repeated sub-culturings on PDA plates of isolated mycelia, pure colonies were obtained and stored in 30% glycerol at −80°C for further study.

To evaluate the growth-promoting activity of the isolated fungus (referred as A3), tobacco seeds were soaked in 10% (v/v) bleach for 15 min, then rinsed with sterilized water four times and finally they were germinated on solid Murashige and Skoog medium (MS). The square petri plates (17 × 17 cm) containing seeds were incubated vertically in the growth chamber for 10 days at 25°C ± 2°C. Then, the isolated fungus (5 μl of a liquid culture) was inoculated at the bottom of treated petri dishes and for control ones 5 μl of sterilized water were added. One month after sowing, the plates were photographed and growth stimulation was evaluated qualitatively basing on root length and leaf surface.

### Microscopic observation and fungal identification

Microscopic observation of A3 was performed as described by Tarroum et al. (2021). In brief, solid PDA medium was cut into small squares, and then transferred to new petri plates containing a wet filter paper. Using sterile needles, the four edges of each PDA block were then inoculated with isolated A3 fungus. Following incubation of plates for 72 h at 27°C, the coverslips were gently removed and transferred to microscope slides with a drop of lactophenol cotton blue stain. The slides were examined and photographed using a Nikon microscope.

Five-day-old mycelium was collected from PDA plate and ground in liquid nitrogen to a fine powder using a mortar and pestle. Genomic DNA was then extracted using a DNeasy Plant Mini Kit (Qiagen) according to the manufacturer’s instructions. The isolated DNA was used for PCR amplification of a nuclear ribosomal internal transcribed spacer (ITS) region which was amplified by universal primers ITS1 (forward) and ITS4 (reverse) as listed in (White et al., 1990). PCR amplification was carried out in a 30 μl reaction volume as described by Tarroum et al. (2021). After running the amplified fragments on 1.5% agarose gels, they were purified and cloned into pGEM^®^-T Easy vectors (Promega) then sequenced by Macrogen Inc., (Seoul Korea). The resulting sequences were used in BLAST searched using NCBI databases to identify the isolated fungus.

### Temperature and salinity effects on A3 growth

To assess the effect of temperature on A3 growth, the mycelium was inoculated on PDA and incubated at 27°C or 40°C (three replicates each). The effect of salinity on fungal growth was tested on MS medium at five NaCl concentrations (100, 200, 400, 800, and 1,000 mM NaCl) with three replicates each. The isolated fungus was sub cultured at the center of 9 cm petri dishes each containing 20 ml medium and incubated at 27°C. The diameter of mycelium growth was measured every 2 days during 15 days.

### Quantification of IAA in A3 filtrate

IAA production was measured according to Gordon and Weber (1951). In brief, the fungus was inoculated in liquid MS medium with or without L-tryptophan (1 mg/ml) and incubated at 27°C for 7, 14, 21, or 28 days. After incubation, each culture was centrifuged at 10,000 rpm during 15 min. The supernatant (1 ml) was mixed with 1 ml of Salkowski reagent (1 ml 0.5 M FeCl3, 30 ml concentrated H_2_SO_4_, and 50 ml distilled H_2_O) and incubated in the dark at room temperature during 20 min. The optical density at 530 nm was measured and the amount of IAA produced was calculated using a standard curve.

### Quantitative evaluation of growth-promoting activity by A3 fungus

To quantitatively evaluate the effects of A3 fungus on plant growth, two vertical plastic boxes were assembled, with the top box containing 15 holes and filled with sterilized vermiculite and fixed to a lower box containing sterilized MS liquid medium. Five sterilized tobacco seeds as described above were germinated into the vermiculite-containing box. Ten days later, five microliters of liquid culture containing isolated A3 fungus added to the MS medium. After three additional weeks of inoculation, plants were harvested and plant height, leaf area, dry weight, and total chlorophyll content were recorded.

### Growth-promoting activity evaluation of A3 cell-free filtrate (CFF)

#### In MS liquid medium

To prepare A3CFF, a mycelium disc of 5 mm in diameter was picked from a 7-day-old culture grown on solid PDA medium then inoculated into a 250 ml Erlenmeyer flask containing 100 ml of MS medium. Cultures were then incubated at 27°C with shaking at 150 rpm for 30 days, vacuum filtered (0.22 μm), and the resulting A3CFF stored at 4°C for further use.

Growth containers were made consisting of two assembled and autoclaved boxes, with the top box containing nine holes into which plants can be inserted and the bottom box filled with 1.5 l of MS liquid media and covered with aluminum foil to avoid algal contamination. Sterilized tobacco seeds were sown in sterile 1.5 ml Eppendorf tubes that were perforated at the bottom and filled with vermiculite. One week after germination, seedlings of similar sizes were selected and transferred to growth containers containing one of three different dilutions of A3CFF (1:500, 1:1000, and 1:2000). Growth containers were then kept in a temperature and humidity-controlled growth chamber. Plants were harvested 1 month later and shoot length, root length; fresh and dry shoot weights, leaf number, and leaf area were recorded. The total chlorophyll and proline content as well as catalase (CAT) and superoxide dismutase (SOD) activity levels were also quantified.

The stability of A3CFF stored for 1 year at 4°C, was evaluated in liquid MS media using the growth containers described above. One week old seedlings were transferred to growth containers supplemented with 1:500 dilutions of fresh or stored A3CFF. The two parts of the growth container were carefully sealed and placed in growth chamber with controlled conditions. One month later, the shoot and root length, dry and fresh weight, leaf number, and leaf area was recorded.

### In NS

In order to examine the potential use of A3CFF in hydroponic growing systems, its ability to promote plant growth when added to a NS in place of chemical fertilizers was evaluated. Full-strength NS was prepared by mixing the following in a total of 1 l water then adjusting the pH to 6.2: 600 mg Chem-GroTM fertilizer 8-15-36, 600 mg CaNO_3_, 373 mg MgSO_4_. Following procedures described above, germinated tobacco seeds were transferred to growth containers in which the bottom part was filled with NS and supplemented with a 1:50 or 1:500 dilution of A3CFF. One month later, morphological parameters such as shoot and root length, fresh/dry weight, leaf number, and leaf area were measured.

### Evaluation of salt stress alleviation of A3CFF

Tobacco seeds were sterilized (as described above) then placed in sterile 1.5 ml Eppendorf tubes (perforated from the bottom) containing vermiculite. The tubes were fixed in a small plastic cups with a hole in the bottom and then kept in a sterile box for germination. Germinated seeds were then transferred to a hydroponic system consisting of two autoclaved boxes, with the top box containing nine holes for plant placement and the bottom box filled with 1.5 l of NS and covered with aluminum foil to avoid algal contamination. After 2 weeks of growth, plants were subjected to one of four treatments: (1) growth in a hydroponic system containing only NS (control), (2) growth in NS supplemented with A3CFF at 1:50 dilution, (3) growth in NS and 250 mM NaCl, (4) growth in NS with 250 mM NaCl and a 1:50 dilution of A3CFF. The boxes containing the plants were kept for 6 weeks in a growth chamber (16 h light, 8 h dark, 25°C ± 2, relative humidity 60–70%). Plants were then harvested, growth parameters listed above recorded, and the samples immediately frozen in liquid nitrogen and kept at −80°C until further use.

### Evaluation of fertilizer input substitution by A3CFF

Sterilized tobacco seeds were placed in sterile 1.5 ml Eppendorf tubes (perforated from the bottom) containing vermiculite. Following 10 days of germination, seedlings were transplanted to a hydroponic growing system (as described above) containing either full (NS) or half (0.5NS) NS. For the treated plants, 1 week after transplantation, the 0.5NS was supplemented with A3CFF at a dilution of 1:50. Plants were grown in a growth chamber (16 h light, 8 h dark, 25°C ± 2, relative humidity 60–70%) and plant growth parameters were measured after 1 month.

### Estimation of physiological and biochemical parameters

#### Chlorophyll

Total chlorophyll was extracted in 80% acetone for 24 h at 4°C in the dark. Absorbance at 645 nm and 663 nm was determined with an Amersham spectrophotometer and used to calculate total leaf chlorophyll content according to the following equation: Total chlorophyll (μg/ml) = 20.2 (A645) + 8.02 (A663) (Liang et al., 2017).

#### Proline

Proline was extracted following the protocol described by Ábrahám et al. (2010). Liquid nitrogen was used to grind 0.5 g of fresh leaf tissue and the resulting powder incubated in 10 ml of 3% aqueous sulfosalicylic acid. The mixture was centrifuged at 10,000 rpm and 2 ml of supernatant added to 2 ml ninhydrin plus 2 ml glacial acetic acid. The mixture was then boiled at 100°C for 1 h and the reaction stopped by transferring the tubes to an ice bath for 5 min. Next, 6 ml of toluene was added, mixed vigorously for 15 s, and the absorbance of the upper phase read at 520 nm. The proline content was expressed in μg/g fresh weight.

#### CAT and SOD activity

Enzymes were isolated as described by Jogeswar et al. (2006). Fresh tissue (100 mg) was ground in liquid nitrogen then incubated in 0.1 M of phosphate buffer (pH 7.4) containing 0.1 mM EDTA, 1% (w/v) PVP, and 0.5% (v/v) Triton-X 100. The homogenate was centrifuged at 14,000 rpm for 20 min at 4°C, and the supernatant was used to estimate CAT and SOD activities.

CAT activity was measured following the method described by Claiborne (1985) and modified by Jogeswar et al. (2006). The reaction mixture consisted of 1 ml 0.06 M H_2_O_2_, 0.1 M sodium phosphate buffer (pH 7.4), 1.9 ml distilled water, and 100 μ enzyme extract. Catalytic activity was recorded by spectrophotomer using the decline in absorbance at 240 nm owing to degradation of H_2_O_2_ and was expressed as units/g protein.

SOD activity was measured following the method described by Chandrakar et al. (2016) with minor modifications. The reaction mixture (3 ml) contained 50 mM of Tris–HCl buffer (pH 8.2), 1 mM EDTA, 100 μl enzyme extract, 0.4 mM pyrogallol, and H_2_O up to 3 ml. SOD activity (U/g protein) was defined as the amount of enzyme required to inhibit 50% of pyrogallol oxidation based on absorbance at 420 nm.

#### Determination of K^+^ and Na^+^ content

The concentrations of Na^+^ and K^+^ were estimated in root and leaf. Dried tissues were incubated in 0.5% HNO_3_ for 7 days (Ben-Romdhane et al., 2018). The supernatant was filtered and the concentration of Na^+^ and K^+^ was then estimated using an atomic absorption spectrometer (Thermo Scientific™, United Kingdom) and reported as mg/g dry weight.

### RNA extraction and genes expression profiling

Total RNA was extracted following plant treatment with A3CFF at three times (0, 24, and 72 h) using the QiagenRNAeasy Plant Mini Kit following the manufacturer’s instructions. After quantification, total RNA was treated with DNaseI (RQ1, Promega, United States). The cDNAs were synthesized as follow: 5 μg of total RNA was reverse transcribed with random hexamer and oligo-(dT18) primers using SuperScript™ III reverse transcriptase (Invitrogen), as described in the manufacturer’s instructions. The cDNA was used for RT-qPCR reactions, which were carried out using 480 SYBER Green I Master Mix (Roche, Switzerland) as described in the manufacturer’s manual. The PCR cycling conditions were as follow: 95°C for 3 min, followed by 40 cycles of 95°C for 20 s, 60°C for 30 s, and 72°C for 1 min. To create a melting curve and check primer specificity, the extension temperature was increased from 72°C to 95°C after 40 cycles of amplification. Actin (380 bp, ACT-F and ACT-R) was selected as a reference gene. Primers targeting salt stress-related genes (NtSOS1, NtNHX1, NtHKT1, NtSOD, and NtCAT1) or those implicated in in nitrogen metabolism (NR1, NRT1), auxin biosynthesis (TRYP1, YUCCA6-like), and brassinosteroid biosynthesis (DET2, DWF4) selected for this study (Supplementary Table S1) were designed *via* primer 3 software.^1^ Target gene expression was quantified and expressed relative to actin expression using either the ΔΔCT comparative method (Ben-Romdhane et al., 2018), ΔΔCt = (Ct target gene - Ct actin) treated plant - (Ct target gene - Ct actin) control or by the semi-quantitative PCR products analyzed using a 1.5% agarose gel. To ensure reproducibility, two biological and three technical replicates were performed for this experiment.

### Statistical analysis

Data were analyzed using one-way ANOVA followed by Duncan’s test *via* SPSS software version 25. Each value presented is the mean of five replicates for all growth parameters and three replicates for qPCR. Different letters on bar charts are used to indicate means that differ significantly at *p* ≤ 0.05.

The identification of isolated fungus was performed by the use of NCBI-BLAST search and the phylogenetic tree was constructed using neighbor-joining method by bootstrapping 1000 times in MEGA X software.

## Results

### Isolation, identification, and qualitative evaluation of fungi growth-promoting activity

**Figure 1:**
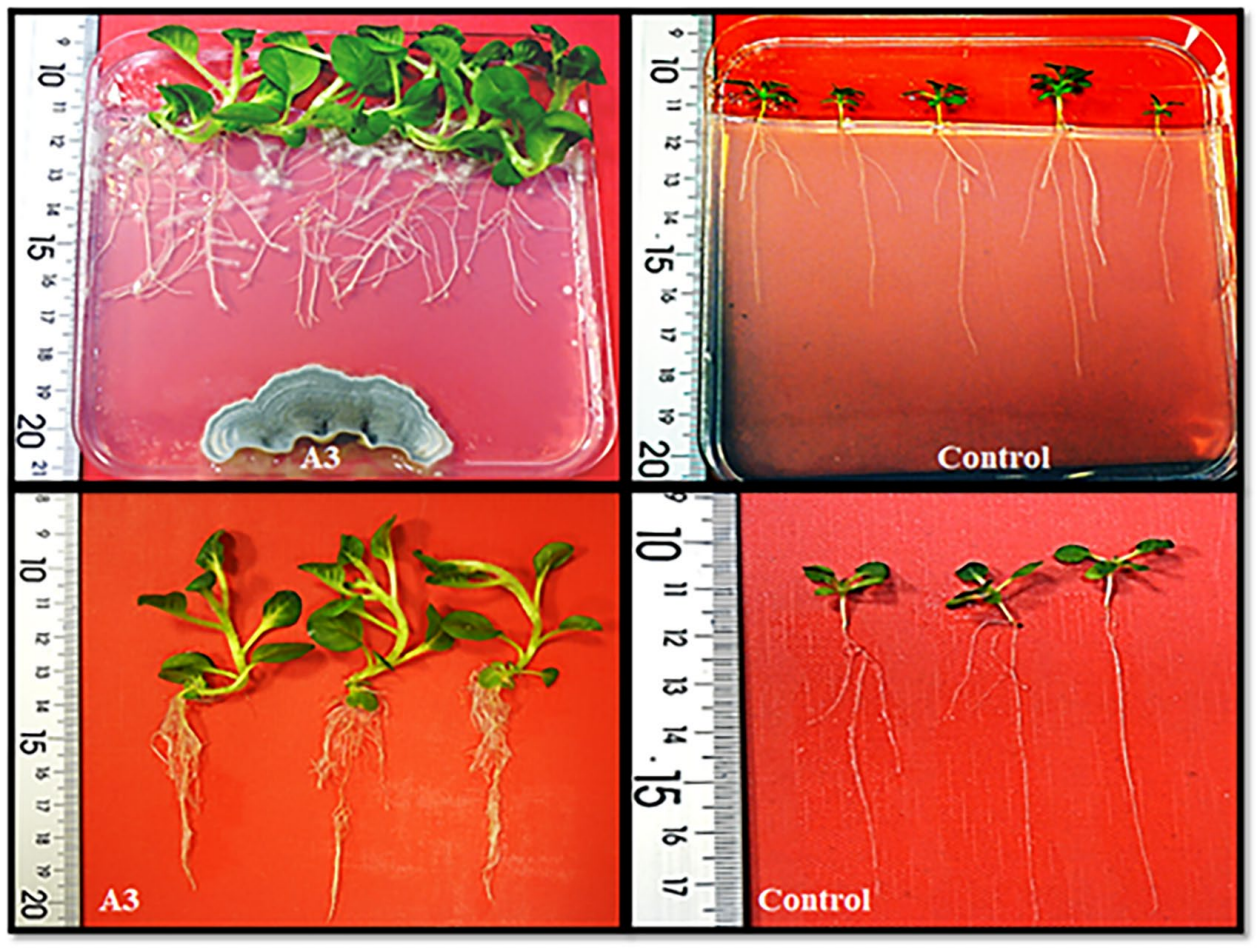
Evaluation of the qualitative growth promoting activity effect of tobacco seedlings cultivated on MS solid medium in the presence of the isolated A3 fungus.

As a first step, 20 soil samples were collected from the rhizosphere of the halophyte grass *A. littoralis* growing in the sebkha of Salboukh. From these samples, 18 fungi (designed A1 to A18) were identified based on visual differences in their morphology when grown on PDA medium. The 18 isolated fungi were evaluated qualitatively for their ability to promote growth of tobacco seedlings cultivated on MS medium. The results showed that out of 18 tested fungi, only seven strains were able to clearly stimulate root and leaf growth of tobacco seedlings (data not shown). For further detailed investigations, the A3 fungus was chosen as it had the greatest effect on plant growth (Figure 1). Morphologically, on PDA media after 1 week of incubation at 27°C, the A3 colonies had moderate growth, with a green color on one side and pale yellow on the reverse side. Microscopic analysis revealed that the A3 strain has a septate hypha terminating in a complex conidiophore. Moreover, chlamydospore was clearly visible at 40x magnification (Supplementary Figure S1a). The growth of A3 showed maximum growth after 12 days of incubation at 27°C. However, at 40°C, total inhibition of A3 growth was observed (Supplementary Figure S1b). Since A3 was isolated from the rhizosphere of the halophyte grass *A. littoralis* its tolerance to NaCl in the culture media was also investigated. Based on mycelial growth levels, the A3 fungus was able to grow in up to 1 M NaCl. Interestingly, the A3 fungus showed the largest colony diameter at 200 mM NaCl, while the growth rate of A3 mycelia was inhibited at NaCl concentrations above 200 mM (Supplementary Figure S1c). Finally, using Salkowski’s reagent, IAA was detected in growth medium containing L-tryptophan at 0.93 ppm after 28 days of incubation. In a medium without L-tryptophan IAA was detected at 0.20, 0.22, 0.24, and 0.26 ppm after 7, 14, 20, and 28 days of incubation, respectively. This confirmed that the addition of L-tryptophan to growth media and extended incubation times are required for maximum auxin production by the A3 fungus (Supplementary Figure S2).

The ITS region was chosen as it is the barcode with the highest probability of correct identifying a huge number of sampled fungi (Raja et al., 2017). The isolated ITS sequence fragment was BLAST searched against the NCBI GenBank and showed 93.4% identity to the one of *Penicillium olsonii* (Supplementary Figure S3). Basing on these results the A3 is likely *P. olsonii*.

### Quantitative evaluation of the A3 growth promoting activity

**Figure 2:**
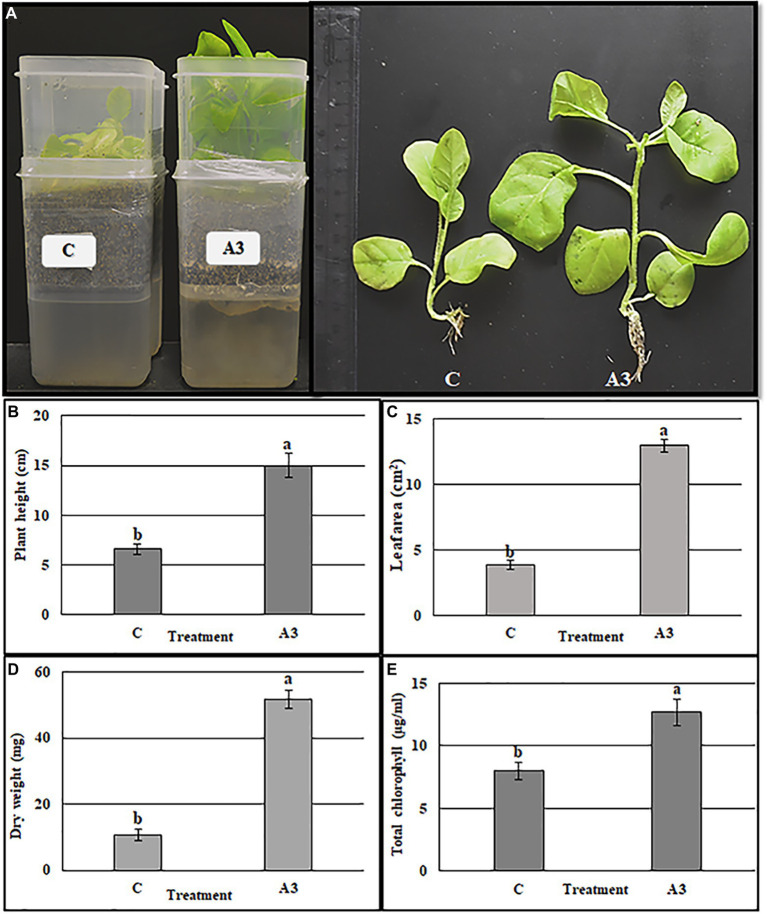
Quantification of A3 growth stimulation activity on tobacco seedling grown in MS liquid medium. Seeds of tobacco were cultivated in the two boxes hydroponic system, vermiculite in the top and MS liquid medium in the bottom. The treatment was performed by adding 5 μl of A3 liquid culture in MS medium after 10 days of sowing seeds. Plant morphology **(A)**, plant height **(B)**, leaf area **(C)**, plant dry weight **(D)**, and total chlorophyll **(E)**: Data are the means of five replicates ± standard deviation; different letters on bars represent the significant values according to Duncan’s test (*p* < 0.05).

The direct effect of A3 mycelium (DE) on plant growth promotion in liquid MS medium was quantitatively evaluated using plastic boxes as described in Materials and Methods (Figure 2A). When compared to control plants, A3 fungus-treated plants showed significantly higher shoot length, leaf area, dry weight, and total chlorophyll content (2, 3, 5, and 1.5 times higher, respectively (Figures 2B–E). These results confirm that the A3 fungus positively enhances growth of tobacco seedlings cultivated in hydroponic systems when added to liquid MS medium and thus A3 is potentially a PGPF.

### Quantitative evaluation of the A3CFF growth promoting activity

#### In MS liquid medium

**Figure 3:**
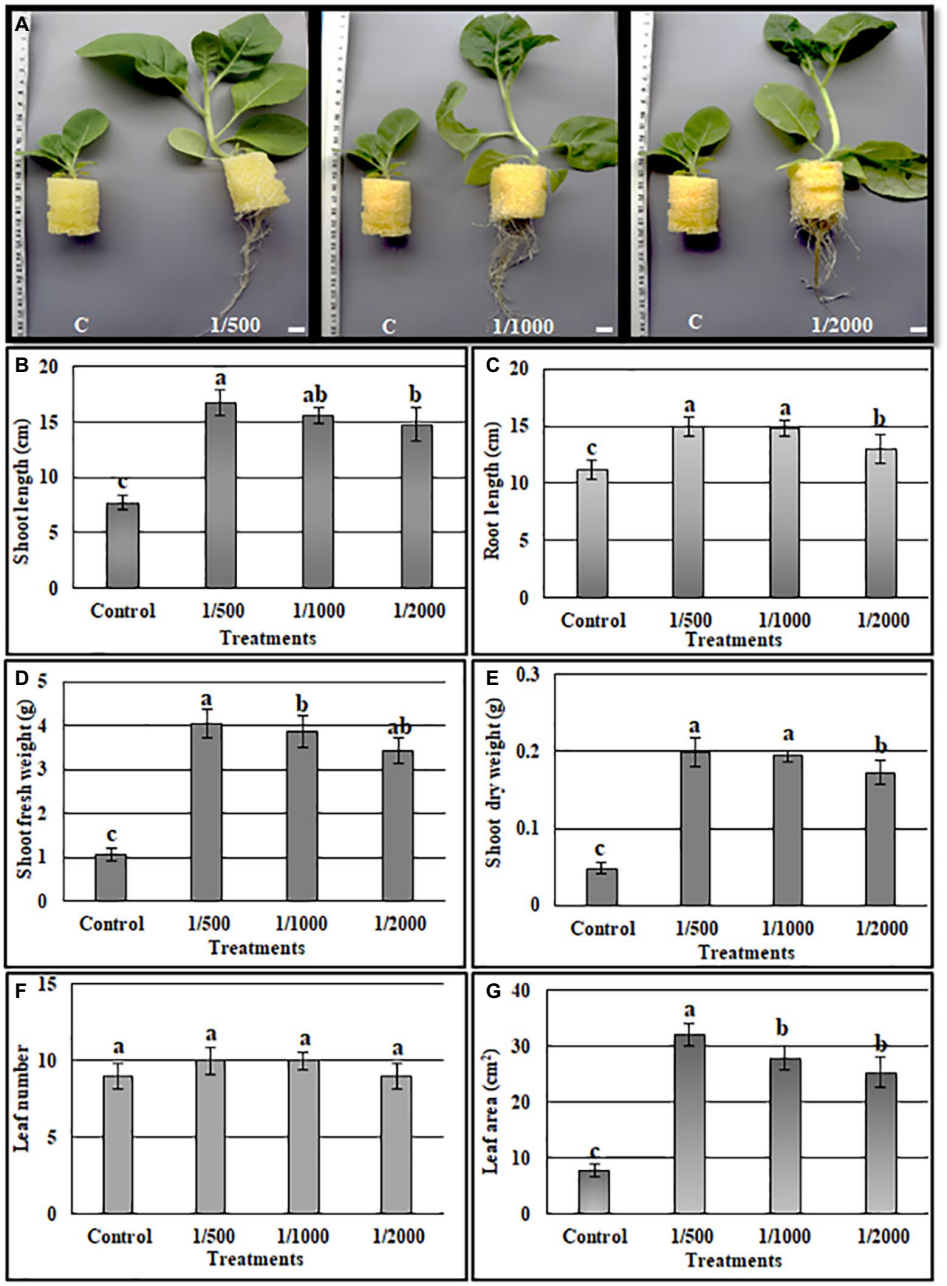
Quantification of A3 cell-free filtrate culture (CFF) growth stimulation activity on tobacco seedling grown in MS liquid medium. The MS liquid medium was supplemented with 1:500, 1:1000 and 1:2000 CFF dilutions. **(A)** Phenotype of plants after 1 month, the shoot length **(B)**, root length **(C)**, shoot fresh weight **(D)**, shoot dry weight **(E)**, leaf number **(F)**, leaf area **(G)**. Data are the means of five replicates ± standard deviation; different letters on bars represent the significant values according to Duncan’s test (*p* < 0.05).

**Figure 4:**
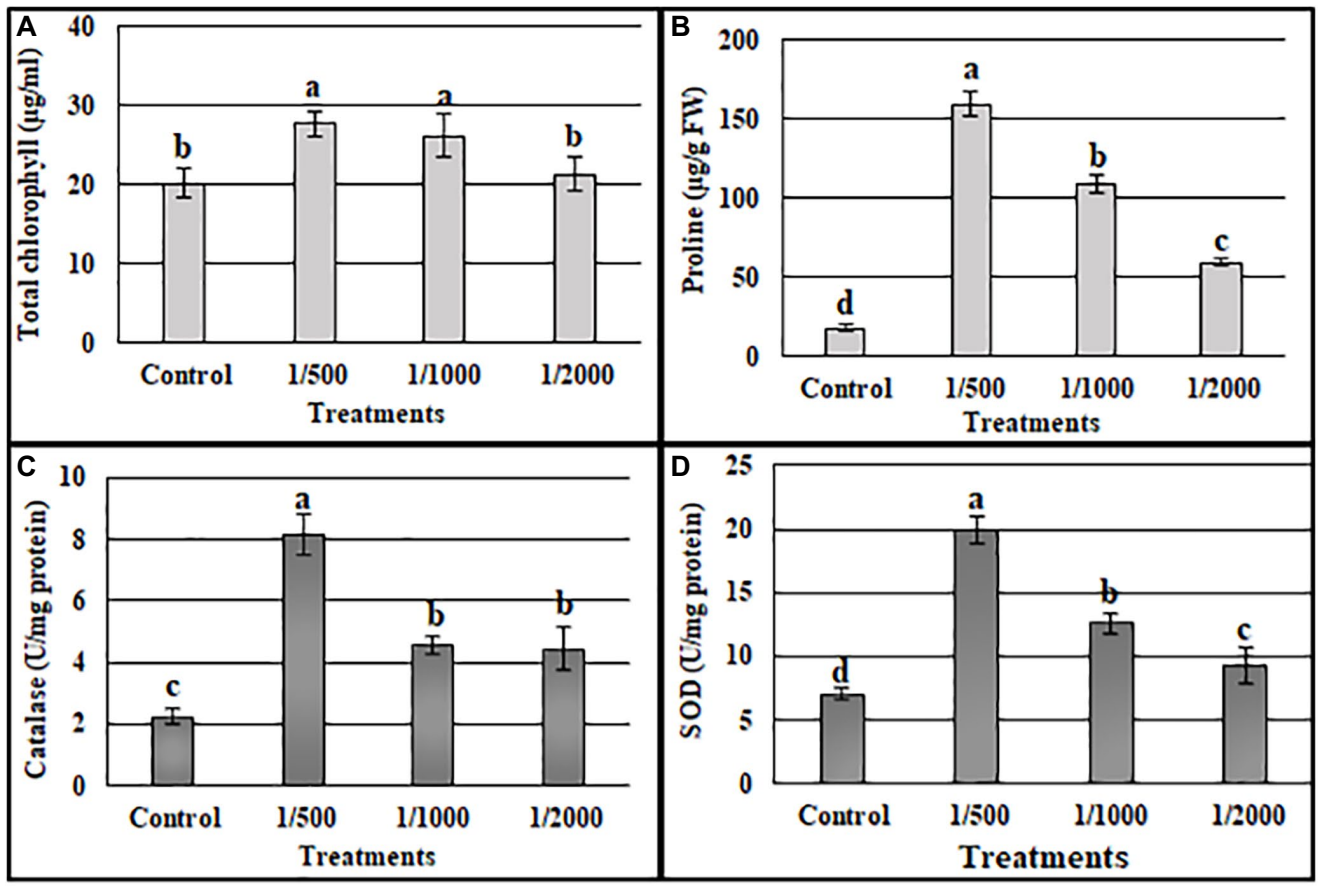
Effects of A3 CFFs at different dilutions (1:500, 1:1000 and 1:2000) on total chlorophyll **(A)**, proline **(B)**, catalase **(C)**, and SOD **(D)** activities in tobacco plants: Data are the means of five replicates ± standard deviation. Different letters on bars represent the significant values according to Duncan’s test (*p* < 0.05).

The ability of cell-free culture filtrate from A3 culture (A3CFF) to stimulate growth of tobacco plants in a hydroponic system was determined. MS liquid medium was used as the growing media and was supplemented with one of three dilutions of A3CFF (1:500, 1:1000, or 1:2000). After 1 month of treatment with A3CFF, net improvements in growth (Figure 3A), physiological (Figures 3B–G), and biochemical (Figure 4) parameters were observed for all three tested dilutions. Each dilution significantly promoted shoot/root length, fresh/dry shoot weight, and leaf area (Figures 3B–G). In addition, the highest values of total chlorophyll (27.7 μg/ml), proline accumulation (159 μg/g FW), as well as CAT (8.1 U/g proteins), and SOD (19.8 U/mg proteins) activities were detected in plants treated with an A3CFF dilution of 1:500 (Figure 4). These increases in proline synthesis and CAT/SOD activities in A3CFF-treated plants suggest that A3 fungus can alleviate salt stress. Finally, we tested the stability of A3CFF by growing tobacco seedlings in liquid MS medium containing a 1:500 dilution of fresh A3CFF or A3CFF stored at 4°C for 1 year (Supplementary Figure S4a). The results demonstrated that the positive effects on plant growth of A3CFF were preserved after 1 year of storage at 4°C (Supplementary Figures S4b–g).

#### In nutritive solution (NS)

**Figure 5:**
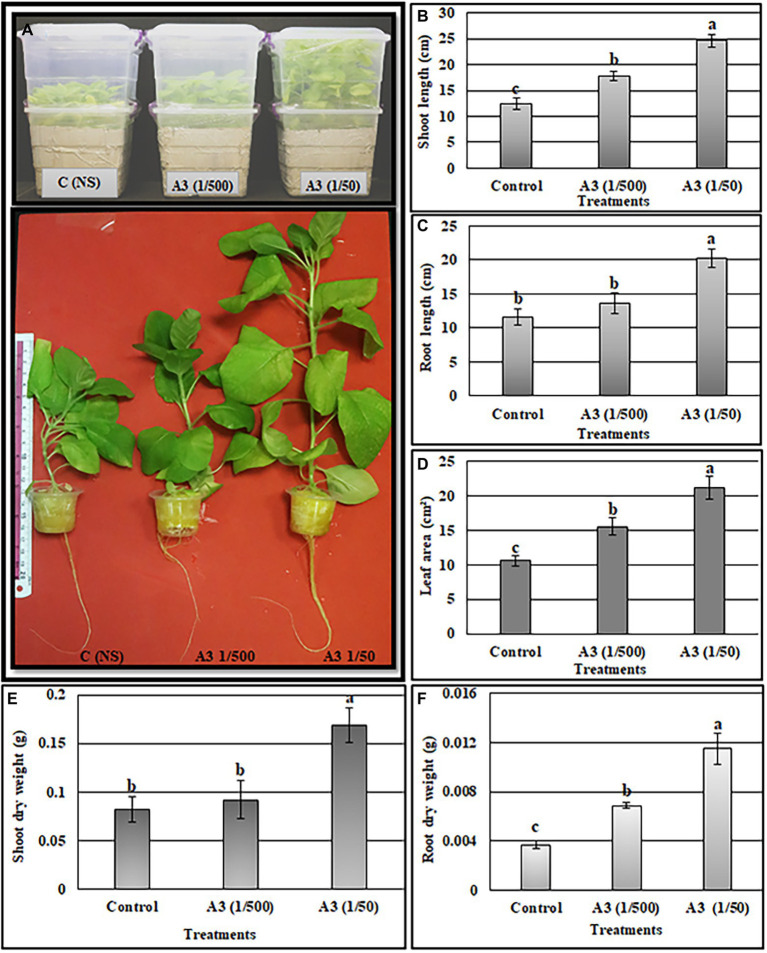
Effects of A3 CFF when added at dilutions of 1/50 and 1/500 to nutrient solution (NS) on promoting tobacco seedlings growth cultivated in hydroponics system. Plant morphology **(A)**, shoot length **(B)**, root length **(C)**, leaf area **(D)**, shoot dry weight **(E)**, root dry weight **(F)**. Different letters on bars represent the significant values according to Duncan’s test (*p* < 0.05).

The above results show that A3CFF stimulated plant growth when added to liquid MS medium in the absence of fungal mycelia. One of our goals from this work was to investigate the effects of PGPF application on hydroponically grown vegetable plants in conditions of reduced fertilization. We thus evaluated the effectiveness of A3CFF in stimulating plant growth when mixed with commercial fertilizers in NS and used in hydroponic system. The two A3CFF dilutions, 1:500 or 1:50, when applied to tobacco seedlings grown in NS significantly stimulated growth (Figure 5A). Interestingly, the A3CFF dilution of 1:50 promoted the highest growth by increasing the shoot/root length, the shoot/root dry weight, and the leaf area by factors of 198, 174, 211, 319, and 200%, respectively (Figures 5B–F).

#### In The presence of salt stress

**Figure 6:**
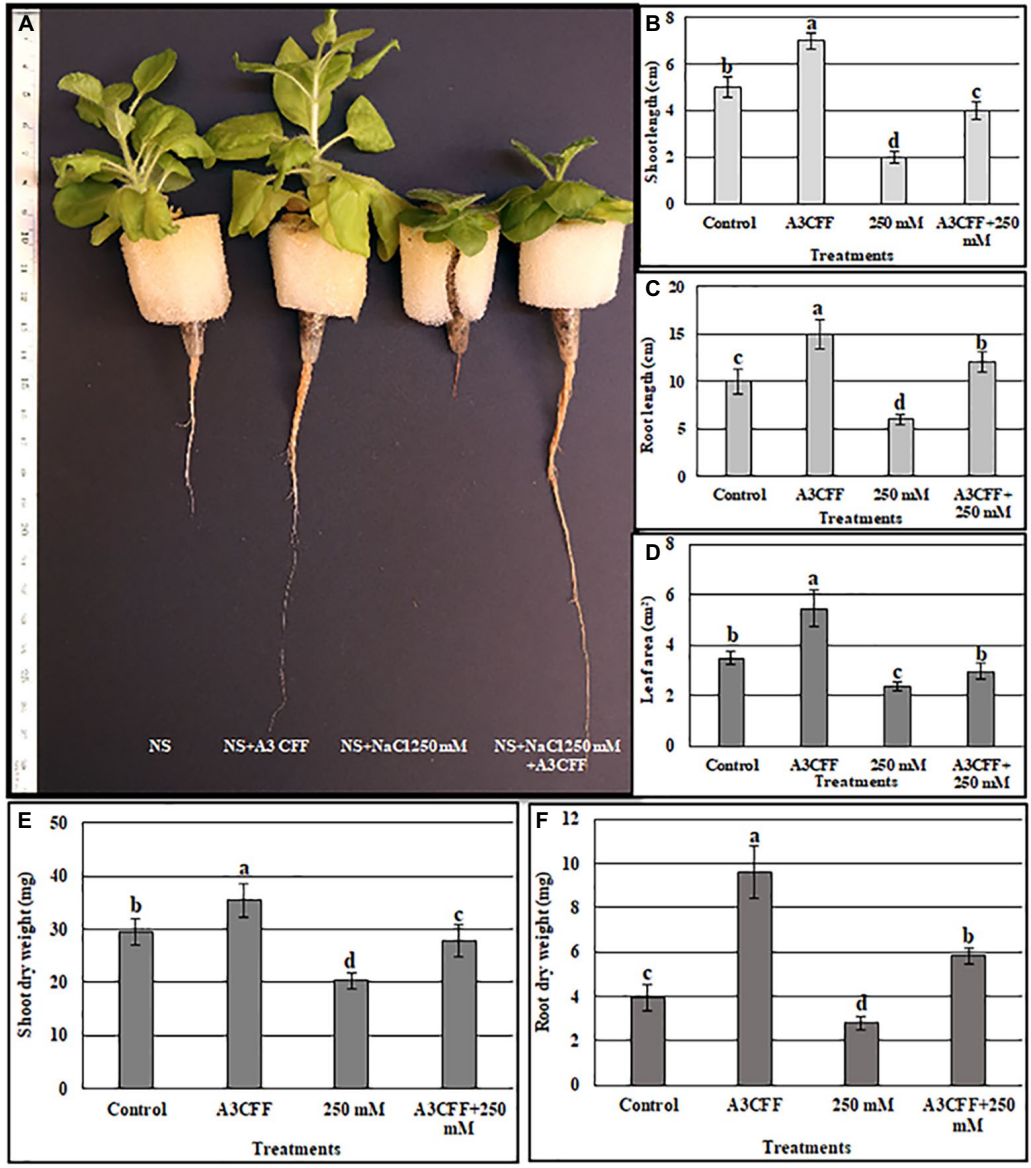
Evaluation the ability of A3 CFF to alleviate salt stress when added at 1:50 dilution to NS. **(A)** plant morphology photographed after 1 month of treatment, Shoot length **(B)**, Root length **(C)**, Leaf area **(D)**, Shoot dry weight **(E)**, and Root dry weight **(F)** of tobacco seedlings grown in NS (control), in NS with CFF (A3CFF), with NaCl (250 mM), with 250 mM NaCl and CFF (A3CFF + 250 mM). Different letters on bars represent the significant values according to Duncan’s test (*p* < 0.05).

Tobacco plants treated with an A3CFF dilution of 1:500 and grown in MS liquid medium produced both higher CAT and SOD activities and proline levels compared to untreated plants (Figure 4). In addition, A3CFF in NS at a dilution factor of 1:50 induced more plant growth. Based on these results, we investigated the ability of A3CFF to alleviate salt stress. The addition of A3CFF to NS at a dilution of 1:50 in the presence or absence of 250 mM NaCl enhanced tobacco plant growth compared to control plants (Figure 6A). The presence of 250 mM NaCl in NS significantly reduced the biomass production shoot/root length, weight, and leaf area of tobacco plants compared to those in NS alone. This growth inhibition due to salt stress was decreased following the addition of A3CFF (1:50) to NS containing 250 mM NaCl and (Figures 6B–F). Additionally, the root lengths of the co-treated plants were significantly larger than that of control plants grown in NS only (Figures 6A,C). Moreover, after 1 month without NaCl, A3CFF increased shoot and root length by factors of 1.4 and 1.5, respectively, compared to control plants.

**Figure 7:**
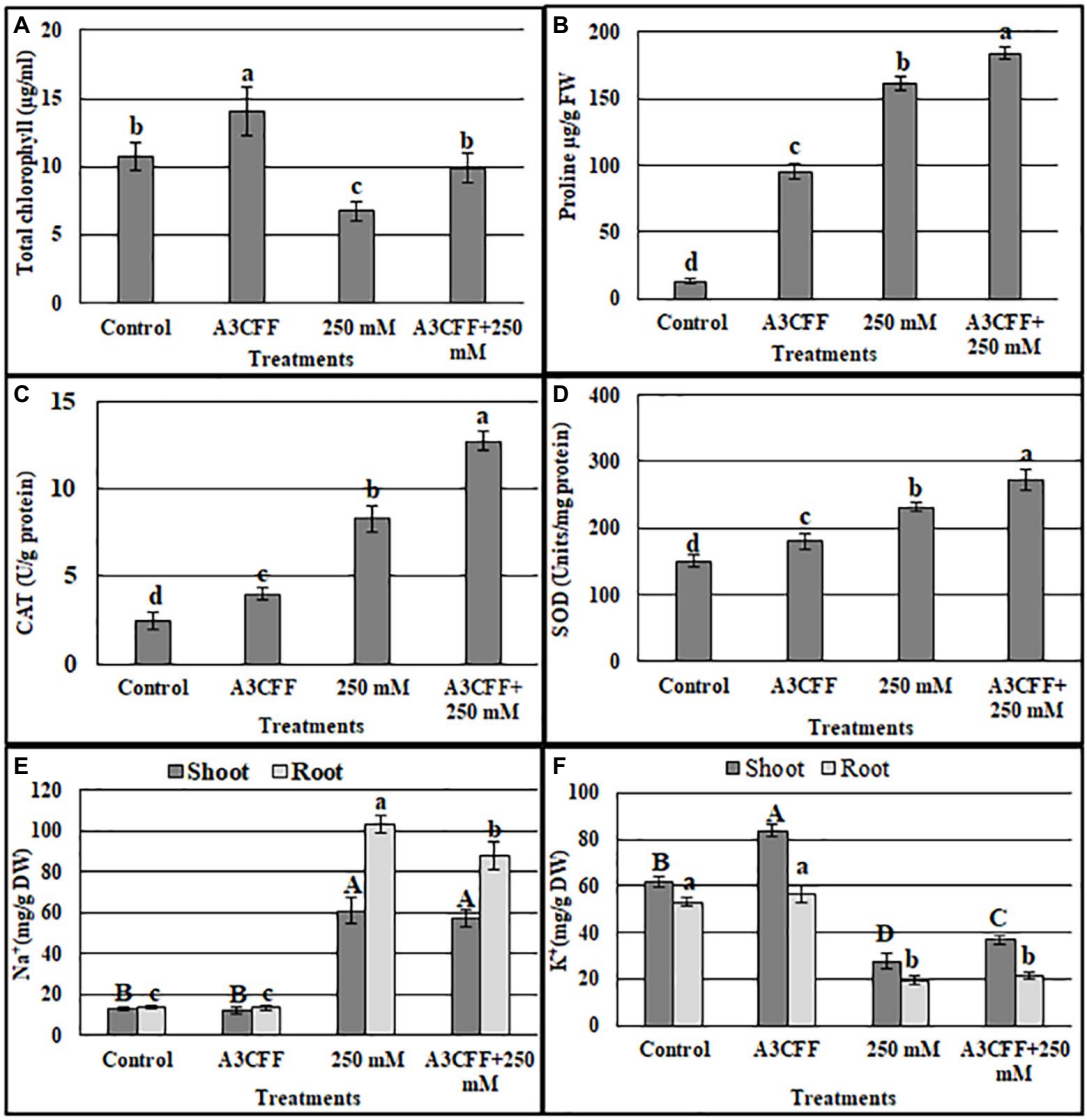
Estimation of total chlorophyll content **(A)**, proline accumulation **(B)**, catalase **(C)**, and SOD **(D)** activities, sodium **(E)** and potassium **(F)** contents in the tobacco seedlings grown in: NS (control), NS with A3 CFF, NS with 250 mM NaCl (250 mM), NS with 250 mM NaCl and A3 CFF (A3CFF + 250 mM). Values are the mean of five replicates ± SD. Different letters on bars represent the significant values according to Duncan’s test (*p* < 0.05).

We also measured additional parameters to further assess the effects of A3CCF on salinity stress responses. Levels of total chlorophyll under salt stress were significantly lower than in control conditions. However, the application of the A3CCF in conjunction with salt stress restored total chlorophyll content to levels comparable to those of control plants under normal conditions (Figure 7A). In addition, the highest value of total chlorophyll, 14.05 μg/ml, was observed in plants grown with A3CCF without NaCl stress (Figure 7A). For proline accumulation, a significant difference was noted between inoculated and non-inoculated plants under salinity stress (Figure 7B). SOD and CAT activities during salt stress were also significantly higher in A3CFF-treated plants compared to non-treated plants (Figures 7C,D).

One of the most important mechanisms for coping with saline toxicity is the reduction of Na^+^ uptake, which is accompanied by the increased K^+^ retention in order to counter balance the Na^+^ entry. Plants treated with 250 mM NaCl showed a significant increase in Na^+^ levels in both roots and shoots compared to the control plants (Figure 7E). The addition of A3CFF to salt-stressed plants decreased Na^+^ accumulation by 14.8% in roots and 6% in shoots compared to control ones. In contrast, in the absence of NaCl, no significant difference in the Na^+^ levels in tissues was detected between inoculated and non-inoculated plants (Figure 7E). Unlike for the Na^+^ levels, salt treatment inhibited the K^+^ uptake in shoots and roots (Figure 7F). Nevertheless, plants inoculated with A3CFF contained significantly more K^+^ compared to non-inoculated plants under both control and salt stress conditions (Figure 7F). Thus these results demonstrated that the A3CFF treatments of plants cultivated in NS with 250 mM NaCl had no effect on Na^+^ and K^+^ concentrations in shoots and roots, respectively. On the other hand, it decreased Na^+^ in roots but increased K^+^ in shoots (Figures 7E,F).

**Figure 8:**
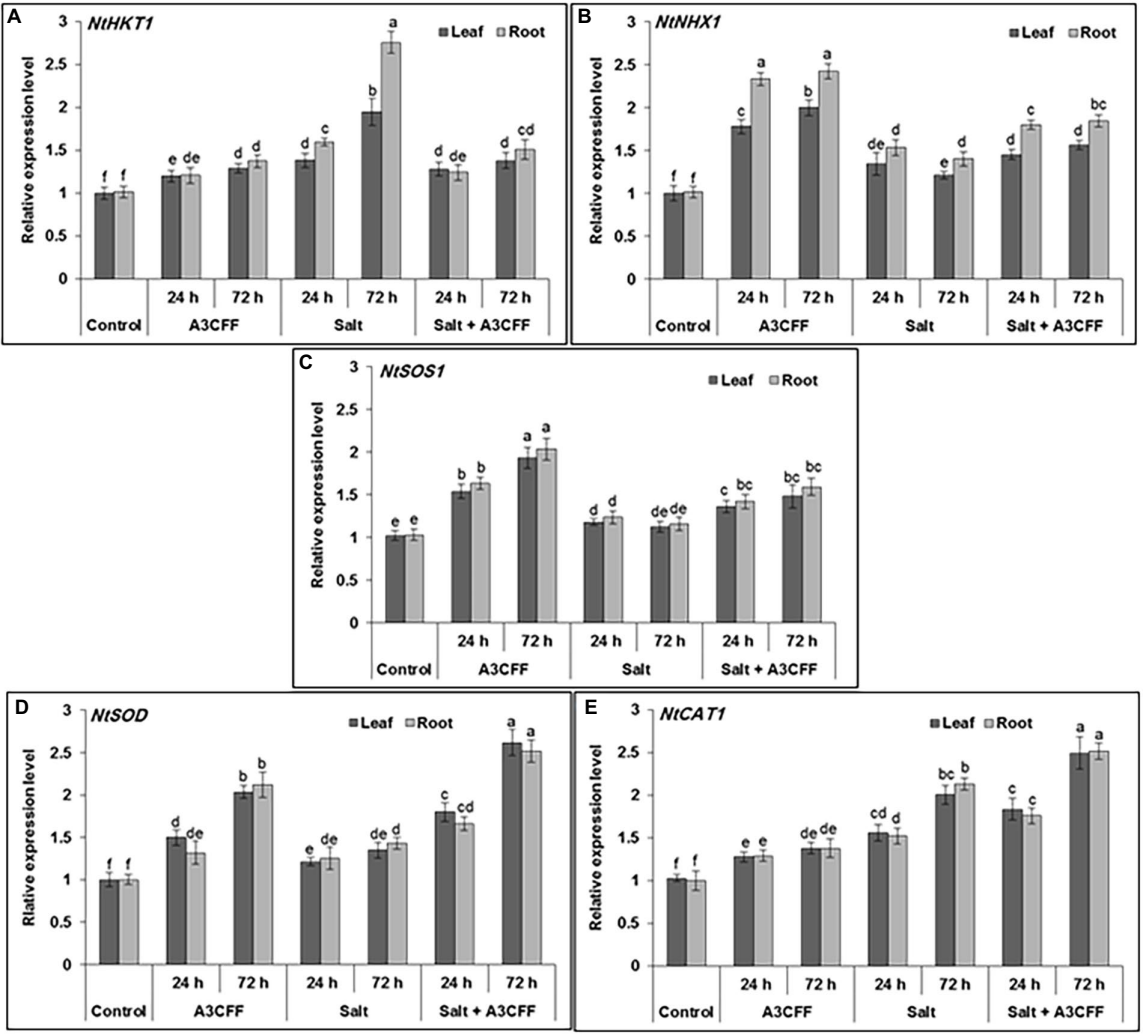
Quantitative RT-PCR analysis showing the effect of A3 CFF on gene expression levels of ROS-related and stress-responsive: HKT1 (XM_015785756), NHX1 (XM_015206776), SOS1 (KY752550), SOD (XM_019401370) and CAT (XM_009795943). The qRT-PCR was performed using RNA extracted from leaf and root at 0, 24, and 72 h of salt (250 mM of NaCl) application. Actin gene was used as reference.

In an attempt to understand the mechanisms by which A3CFF helps plants to cope with high salinity, the expression profiles of multiple salt stress genes (NtHKT1, NtNHX1, NtSOS1, NtSOD, and NtCAT1) were investigated in plants treated with A3CFF and 250 mM NaCl. Adding A3CFF to NS with or without 250 mM NaCl resulted in clear and significant upregulation in both roots and leaves of NtSOD and NtCAT1, which are implicated in the antioxidative mechanisms (Figures 8D,E). The highest expression levels of these two genes were recorded in the leaves and roots 72 h after the addition of A3CFF to NS with 250 mM NaCl. The other three salt stress-associated genes examined, NtHKT1, NtNHX1, and NtSOS1, are the key determinants of Na^+^ and K^+^ homeostasis in plant cells and were upregulated following A3CFF treatment (Figures 8A–C). The Na^+^/H^+^antiporters (NtNHX1 and NtSOS1), which help plants to avoid Na^+^ build-up in shoots were significantly upregulated in A3CFF-treated plants grown in NS with 250 mM NaCl compared to those cultivated only in the presence of salt stress. Moreover, this accumulation was higher in roots compared to leaves (Figures 8B,C).

### Evaluation of A3CFF on chemical fertilizer inputs

**Figure 9:**
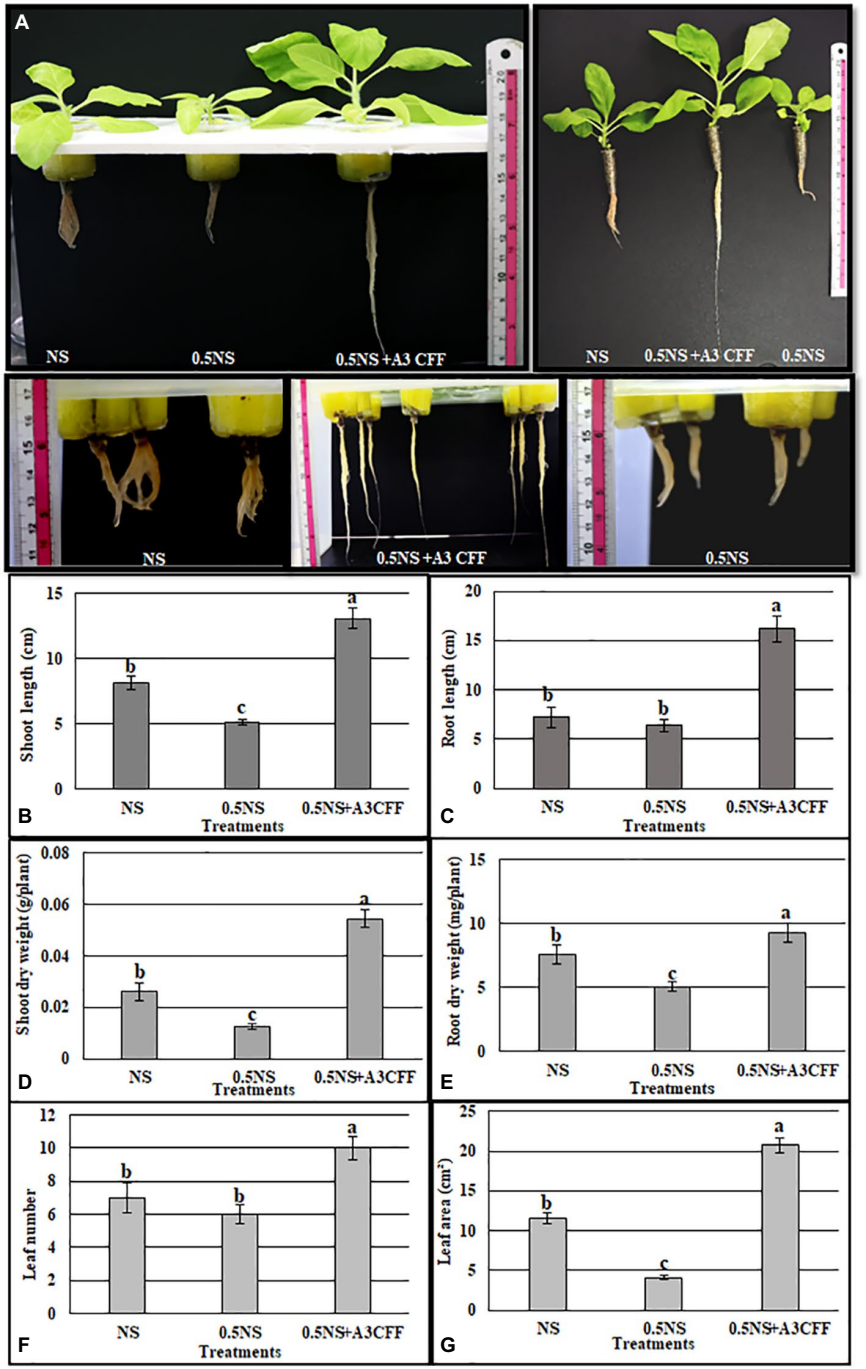
Effect of A3 CFF on tobacco plant growth cultivated in hydroponic system and in the presence of half strength NS (0.5NS). **(A)** Phenotypes of tobacco seedlings grown hydroponically in full NS, in 0.5 NS, in half NS supplemented with 1:50 dilution of A3 CFF (0.5NS + 1:50CFF). The photos were taken after 1 month following treatments. Shoot length **(B)**, root length **(C)**, shoot dry weight **(D)**, root dry weight **(E)**, leaf number **(F)**, and leaf area **(G)** in tobacco seedlings grown in 0.5 NS. Values are the mean of five replicates ± SD. Different letters on bars represent the significant values according to Duncan’s test (*p* < 0.05).

The effectiveness of A3CFF in decreasing chemical fertilizer inputs without affecting plant growth was investigated by growing plants in 0.5NS supplemented with 1:50 dilution of A3CFF, 0.5NS alone, or full-strength NS (Figure 9A). Plants cultivated in 0.5NS and 1:50 A3CFF showed significantly enhanced growth parameters compared to plants cultivated only in NS or 0.5NS. Specifically, shoot/root length, dry weight, leaf number, and leaf area of plants grown in 0.5NS with 1:50 A3CFF were 160, 225, 209, 123,142, and 180%, respectively, of those values for plants grown in full NS (Figures 9B–G). These results revealed that A3CFF was able to compensate for the decrease in NS to half strength, suggesting that A3CFF can minimize the chemical fertilizers required for hydroponic systems without harming plant growth.

The effects of the A3CFF on expression levels of several genes involved in plant growth, including DET, DWF4 (brassinosteroid biosynthesis), YUCCA6-like, TRYP1 (auxin biosynthesis), and NR1 and NRT1 (nitrogen-use efficiency) were examined in tobacco leaves and roots. Transcripts of all six genes were upregulated in both leaves and roots after A3CFF application (Supplementary Figure S5). The greatest transcript accumulation was observed for the YUCCA6-like and NR1 genes, which were most upregulated after 48 h and 72 h of treatment. Based on our results, we propose that A3CFF can induce the expression of growth-regulating DET2, DWF4, YUCCA6-like, and TRYP1 genes, likely explaining the observed plant growth stimulation. Moreover, accumulation of transcripts for the nitrogen-use efficiency-related genes NR1 and NRT may explain the compensation of A3CFF for decreased fertilizer levels in 0.5NS media.

## Discussion

The main goals of smart agriculture are to assure both the quality and quantity of crops ‘yield by developing organic and sustainable production systems. For this, the development of hydroponic systems ensures the reduction of fertilizer inputs and water consumption. Also, biostimulants are used in combination with conventional fertilizers to improve nutrient use efficiency and/or quality of crops (Halpern et al., 2015). The plant rhizosphere constitutes a valuable source of beneficial microorganisms that play important roles in shoot and root growth enhancement. PGPFs directly and indirectly influence both growth and productivity of a wide range of host plants with one study estimating that approximately 44% of rhizosphere fungal isolates are PGPFs (Hossain et al., 2017). In our study, a fungus designated *P. olsonii* was isolated from the rhizosphere of the extremophile plant *A. littoralis*. This fungus was found to significantly promote plant growth in solid or liquid culture media. Based on morphological and microscopic observations, the A3 fungus was identified as a *Penicillium* species, the morphological characteristics were in agreement with *Penicillium* description reported by Frisvad and Samson (2004). The ITS region has previously been shown to be an efficient tool for identifying huge numbers of fungal species and can help accommodate barcode gaps between inter and intra specific variation (Schoch et al., 2012). Furthermore, within the genus *Penicillium*, ITS marker has been noted to work well for providing species information (Visagie et al., 2014). Basing on the ITS sequences, the A3 fungus was identified as *P. olsonii.*

Our results showed that the optimum growth of A3 fungus is at 27°C but completely inhibited at 40°C. This finding is consistent with Diaz et al. (2002), who reported that *P. olsonii* grows at temperatures between 10°C and 25°C. In addition, the growth of *Penicillium marneffei* is dramatically inhibited at 40°C (Cao et al., 2007). As the A3 was isolated from salty soil we investigated its tolerance to the presence of NaCl in growth media. The results showed that A3 fungus tolerated up to 1,000 mM NaCl and it is an obligate halophile strain since its greatest growth was observed in the presence of 200 mM NaCl. This result is consistent with previous reports, as the growth of *P. olsonii* isolated from a sausage was enhanced by adding 3% NaCl to the media (Diaz et al., 2002). Moreover, previous research demonstrated that *P. spathulatum* grows in medium containing 5% NaCl (Frisvad et al., 2013).

Fungal culture filtrates can contain metabolites, which are chemicals produced by fungi *via* multi-step enzymatic pathways. These metabolites include amino acids, mycotoxins, cyclic aromatic peptides, phenols, terpenoids, and plant growth regulators (Ogórek, 2016). In this study, we demonstrated that the A3 fungus secreted auxin, especially when the culture media was enriched with L-tryptophan. Several fungal species that interact with plants have been revealed to produce and secrete auxin (Chanclud and Morel, 2016). The endophyte *Penicillium* sp. isolated form the Halophyte plants was reported to produce IAA (Kondrasheva et al., 2022). Similarly, *Phoma glomerata* and *Penicillium menonorum* can synthesize IAA, which may cause the significant growth increases detected in cucumber plants cultivated with both species (Babu et al., 2015; Waqas et al., 2015).

The quantitative evaluation of plant growth promotion by the A3 fungus was initially performed by directly applying A3 mycelia to liquid MS culture media. The most important value reflecting the growth-promoting activity of A3 fungus is the dry biomass production, which is 4.8 times higher compared to control plants. Similarly, *Penicillium menonorum*, *Penicillium citrinum*, and *Penicillium simplicissimum* were reported to increase the growth of cucumber, sunflower, and *A. thaliana* plants, respectively (Hossain et al., 2007; Babu et al., 2015; Waqas et al., 2015).

Cell-free culture filtrates produced by PGPFs have been found to effectively stimulate plant growth (Hossain et al., 2017), therefore we examined effects of A3CFF from cultures on plant growth. All tested dilutions (1:500, 1:1000, 1:2000) in MS liquid medium, resulted in significant increases of tobacco seedling growth parameters. Moreover, biochemical analyses of plants grown in all A3CFF dilutions revealed high levels of chlorophyll, proline, high SOD and CAT activities. The highest growth effects were observed by using a dilution factor of 1:500 of A3CFF. Moreover, it was demonstrated that the A3CFF activity was conserved even following storage at 4°C for 1 year. To our knowledge, this represents a novel investigation of CFF characteristics. This is very important result for applied practices to integrate the A3CFF into nutritive solutions for hydroponics system. Based on these results, the effects of two concentrations of A3CFF (1:50, 1:500) on plant growth in NS were examined. Only the 1:50 dilution of A3CFF significantly enhanced all growth parameters of tobacco seedlings. Similarly, the CFF from *Penicillium citrinum* cultures induces growth stimulation when applied to *Atriplex gemelinii* seedlings cultivated in solid agar medium, this was attributed to the secondary metabolites secreted by this fungus (Khan et al., 2008). In addition, the application of filtrate from *Penicillium simplicissimum* GP17-2 cultures was as effective as living *Penicillium* in promoting the growth of Arabidopsis. In contrast, while direct application of *Penicillium* sp. GP16-2 promoted plant growth, CFFs from *Penicillium* sp. GP16-2 cultures failed to enhance growth (Hossain et al., 2007, 2008). Besides improving plant growth, it was found that the cell-free culture filtrate from the PGPF *P. indica* cultures increased callus growth and induced lignin production (Kumar et al., 2012; Baishya et al., 2015).

As described above, A3CFF diluted in NS enhanced proline synthesis as well as SOD and CAT activities in tobacco seedlings grown in MS liquid medium. Several studies have demonstrated that proline is involved in osmotic homeostasis adjustments and that antioxidant enzymes such as SOD and CAT are important components of salt tolerance mechanisms in plant (Kumar et al., 2018; Ghosh et al., 2022). Basing on these findings, we tested the ability of A3CFF to alleviate salt stress in tobacco seedlings. Effects of salt stress were evident for tobacco plants grown in NS containing 250 mM NaCl, however, the addition of a 1:50 dilution of A3CCF increased all growth parameters relative to control plants. These results could be explained by the presence of IAA in the A3CFF and the significant increases in activities of SOD, CAT and levels of proline biosynthesis in treated plants. To develop sustainable approaches for salt stress alleviation, many beneficial microorganisms have been used that are efficient, low-cost, and readily adaptable (Fan et al., 2016). The harmful effects of excess salt not only disturbs ionic homeostasis and water uptake, but also causes inadequate oxidative stress responses and imbalances of growth-regulating hormones (Khalid and Aftab, 2020). The auxin secreted by cultured fungi may thus play a major role in adjusting the hormonal balance of stressed plants, contributing to stress alleviation. The application of *Phoma glomerata* and *Penicillium* sp. cultures containing IAA to cucumber plants significantly increased biomass and related growth parameters during NaCl and polyethylene glycol stresses (Waqas et al., 2012). On a related note, the IAA-producing fungus *Aspergillus aculeatus* accelerates the growth of bermudagrass under salt stress (Xie et al., 2017). It has also been reported that the endophytic fungus *Yarrowia lipolytica* promotes the growth of salt-stressed maize plants by controlling plant metabolism and hormonal (IAA and ABA) secretions (Jan et al., 2019). Likewise, application of the PGPFs *Trichoderma longibrachiatum*, *Trichoderma harzianum*, and *Paecilomyces formosus* promoted the growth of wheat, *Suaeda salsa* L, and cucumber plants, respectively, under high salinity conditions (Khan et al., 2012; Chen et al., 2016; Zhang et al., 2016).

Environmental stresses often reduce the chlorophyll content of plants, while chlorophyll accumulation is considered a potential indicator of salinity tolerance (Shah et al., 2017). In the present study, it was determined that the A3CFF significantly increased the chlorophyll content of tobacco seedlings under both normal and salinity stress conditions. Under salt stress conditions, Khan et al. (2011) observed that the growth, chlorophyll content, and photosynthesis rates were significantly higher in plants treated with *Penicillium funiculosum* LHL06 than in untreated plants. In the same way, the chlorophyll content was found to be higher in salt-stressed maize seedlings that had been inoculated with *Yarrowia lipolytica* than in uninoculated plants (Jan et al., 2019). Proline is a low molecular weight amino acid that is a well-known as osmoregulator and ROS scavenger for the salt stressed plants(Bhuyan et al., 2019; Tisarum et al., 2020). Treatment with *Trichoderma longibrachiatum* and *Trichoderma harzianum* has been found to significantly increase the proline levels in salt-stressed wheat and *Brassica juncea* L plants, respectively (Ahmad et al., 2015; Zhang et al., 2016). In our study, under control conditions, the A3CFF was observed to exert no effect on the Na^+^ accumulation in plants (shoots and roots), although it significantly increased the K^+^ content of leaves. However, under salinity stress conditions, the A3CFF significantly decreased the Na^+^ content of roots and increased the K^+^ accumulation in leaves. It has previously shown that endophytic symbiotic fungi can both improve nutrient assimilation and assist in ionic homeostasis maintenance in saline-stressed plants (Gupta et al., 2020). For instance, the colonization of *A. thaliana* with *P. indica* under salt stress has been found to lower the Na^+^/K^+^ ratio (Abdelaziz et al., 2017). Similarly, the saline stress of *Suaeda salsa* L was determined to be counteracted by *Trichoderma harzianum* T83 colonization, thereby resulting in a higher K^+^ uptake (Chen et al., 2016).

In the experiments conducted in this study, it was demonstrated that theA3CFF induced the growth of roots under salt stress, a finding that may be explained by the low Na^+^/K^+^ ratio observed in such plants. The analysis of the expression profiles of NtCAT1 and NtSOD confirmed the biochemical experimental results describing the CAT and SOD activity levels. Indeed, the A3CFF induced the upregulation of NtCAT1 and NtSOD in both the presence and absence of NaCl. Moreover, the genes encoding the key Na^+^ transporters (NtSOS1, NtNHX1, and NtHKT1) involved in salt tolerance were also shown to be upregulated by the A3CFF treatment. Based on these results, it is likely that the enhanced salt tolerance observed in the presence of A3CFF at 250 mM NaCl occurred *via* the regulation of Na^+^ homeostasis by the Na^+^ transport system control at the whole-plant level. In addition, the enhancement of the antioxidant activities (CAT and SOD) may also have helped the plants to endure salinity stress.

It has been reported that plant growth-promoting microorganisms are able to increase the nutrient use efficiency of plants. This effect allows for crop production without yield loss in NS with 50% lower fertilizer inputs compared to in full NS (Da Costa et al., 2013). Our work revealed that reducing NS strength by 50% did not decrease plant growth when the NS was supplemented with a 1:50 dilution of A3CFF. Compared to plants grown in full NS, plants grown in 0.5NS showed significantly reduced shoot length, shoot dry weight, and root dry weight, and leaf area, while the addition of A3CFF significantly increased these parameters. In order to ultimately develop sustainable agricultural practices, several studies have been performed that use PGPFs to decrease the chemical inputs required for maximal yield (Xia et al., 2019). In one such study, tomatoes cultivated in pots or in field trials that were treated with 25% of typical commercial fertilizer levels plus *Trichoderma*-enriched bioorganic fertilizer produced yields equivalent to tomato plants treated with normal amounts of chemical fertilizer (Ye et al., 2020).

In an attempt to explain the mechanisms underlying the promotion of plant growth by A3CFF, the effects of the A3CFF treatment on the accumulation of transcripts for six genes related to growth, brassinosteroid biosynthesis (DET2, DWF4), auxin biosynthesis (YUCCA6-like, TRYP1), and nitrogen-use efficiency (NR, NRT1) were examined. All six genes were found to be upregulated in both roots and leaves following the addition of the A3CFF to the growth media. To our knowledge, there have been no previous reports showing a role for *P. olsonii* activation of genes related to plant growth. However, other species of PGPFs have been shown to influence growth-related gene expression, as *P. indica* was able to stimulate the expression of auxin-responsive genes and nitrate reductase-encoding genes in Arabidopsis and tobacco seedlings (Sherameti et al., 2005; Meents et al., 2019). Similarly, inoculation with *Trichoderma* increased accumulation of auxin-regulated gene transcripts in Arabidopsis and triggered ethylene/indole-3-acetic acid signaling in tomato plants (Contreras-Cornejo et al., 2009; De Palma et al., 2019). However, our study is the first report demonstrating the effect of A3CFF on genes involved in brassinosteroid biosynthesis.

## Conclusion

In conclusion, this study isolated a fungus (A3) from the rhizosphere of the halophyte grass *A. littoralis*, identified as *P. olsonii*, and for the first time, described its role as a PGPF. This strain was determined to significantly promote tobacco plant growth in MS and NS, both directly *via* the application of A3 mycelium and through the application of its CFF. In addition, the A3CFF appeared stable following storage at 4°C for 1 year. The application of the A3CFF decreased the chemical fertilizer levels in NS by 50% and also significantly increased plant growth when compared with the control conditions. Finally, the A3CFF was found to mitigate salinity stress by inducing morphological, biochemical, and molecular changes in salt-stressed plants. Thus, the A3CFF could be used in hydroponics as a promising biotechnological tool for enabling the reduction of expensive chemical fertilizer inputs without compromising the yield, thereby improving agriculture and sustainability.

## Data availability statement

The original contributions presented in the study are publicly available. This data can be found at: NCBI, OP680782.

## Author contributions

MT, LF, and AH: conceptualization, methodology, writing—review and editing. MT, WR, and AH: formal analysis. AA, FA-Q, and AA-D: investigation. MT: writing—original draft preparation. AH: visualization. LF and AH: supervision. All authors have read and agreed to the published version of the manuscript criteria.

## Funding

This research was funded by (RSP-2021/73) at King Saud University, Riyadh, Saudi Arabia.

## Conflict of interest

The authors declare that the research was conducted in the absence of any commercial or financial relationships that could be construed as a potential conflict of interest.

## Publisher’s note

All claims expressed in this article are solely those of the authors and do not necessarily represent those of their affiliated organizations, or those of the publisher, the editors and the reviewers. Any product that may be evaluated in this article, or claim that may be made by its manufacturer, is not guaranteed or endorsed by the publisher.

## Footnotes

^1^ http://bioinfo.ut.ee/primer3-0.4.0/
