# Supplementary material for: A novel PGPF Penicillium olsonii isolated from the rhizosphere of Aeluropus littoralis promotes plant growth, enhances salt stress tolerance, and reduces chemical fertilizers inputs in hydroponic system
Source: Front Microbiol. 2022 Oct 27;13:996054. doi: 10.3389/fmicb.2022.996054 (PMC9648140; doi:10.3389/fmicb.2022.996054)
Supplement: Supplementary file 1 [file Presentation_1.PPTX]

## Slide 1
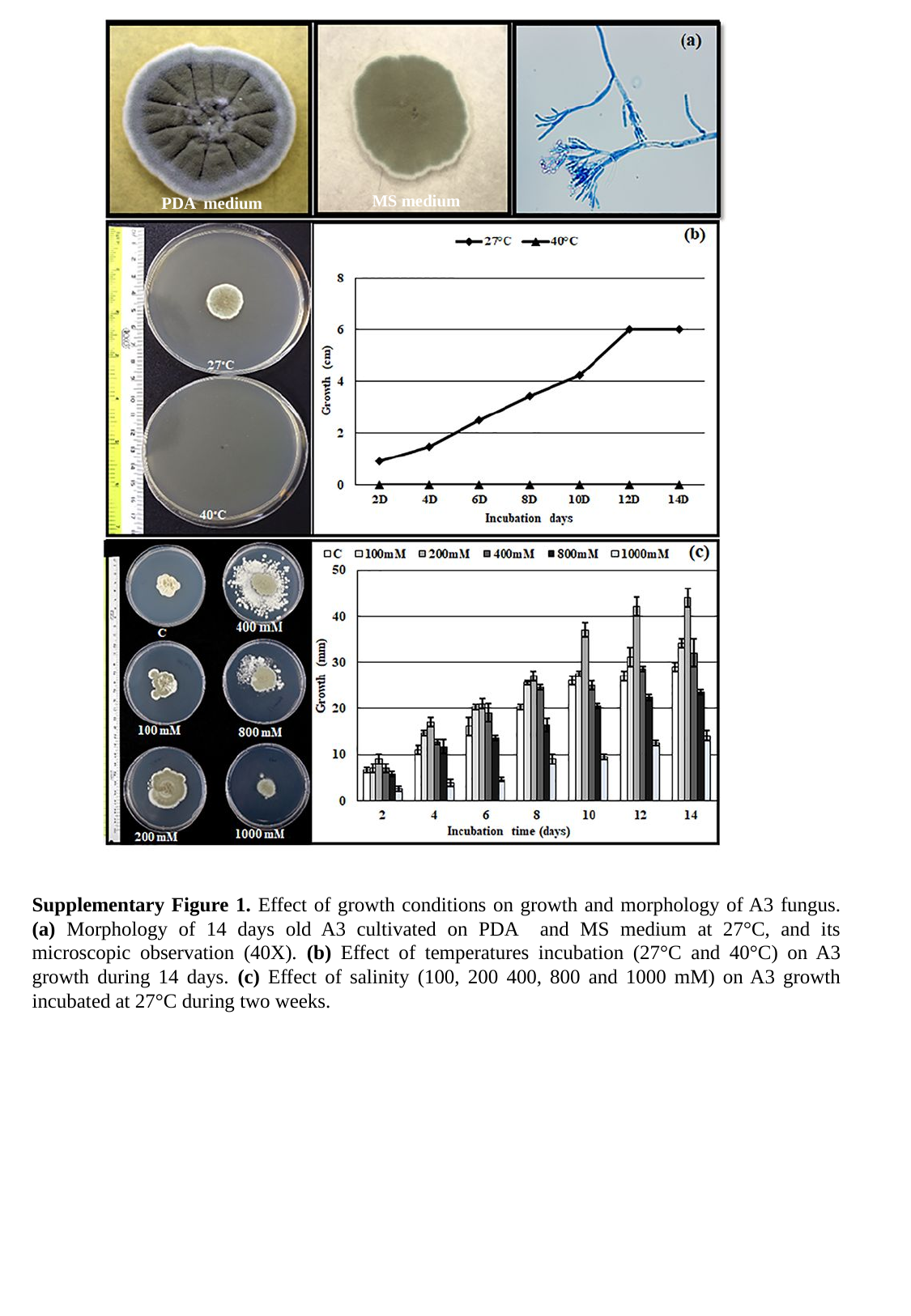

MS medium
PDA medium
Supplementary Figure 1. Effect of growth conditions on growth and morphology of A3 fungus. (a) Morphology of 14 days old A3 cultivated on PDA and MS medium at 27°C, and its microscopic observation (40X). (b) Effect of temperatures incubation (27°C and 40°C) on A3 growth during 14 days. (c) Effect of salinity (100, 200 400, 800 and 1000 mM) on A3 growth incubated at 27°C during two weeks.

## Slide 2
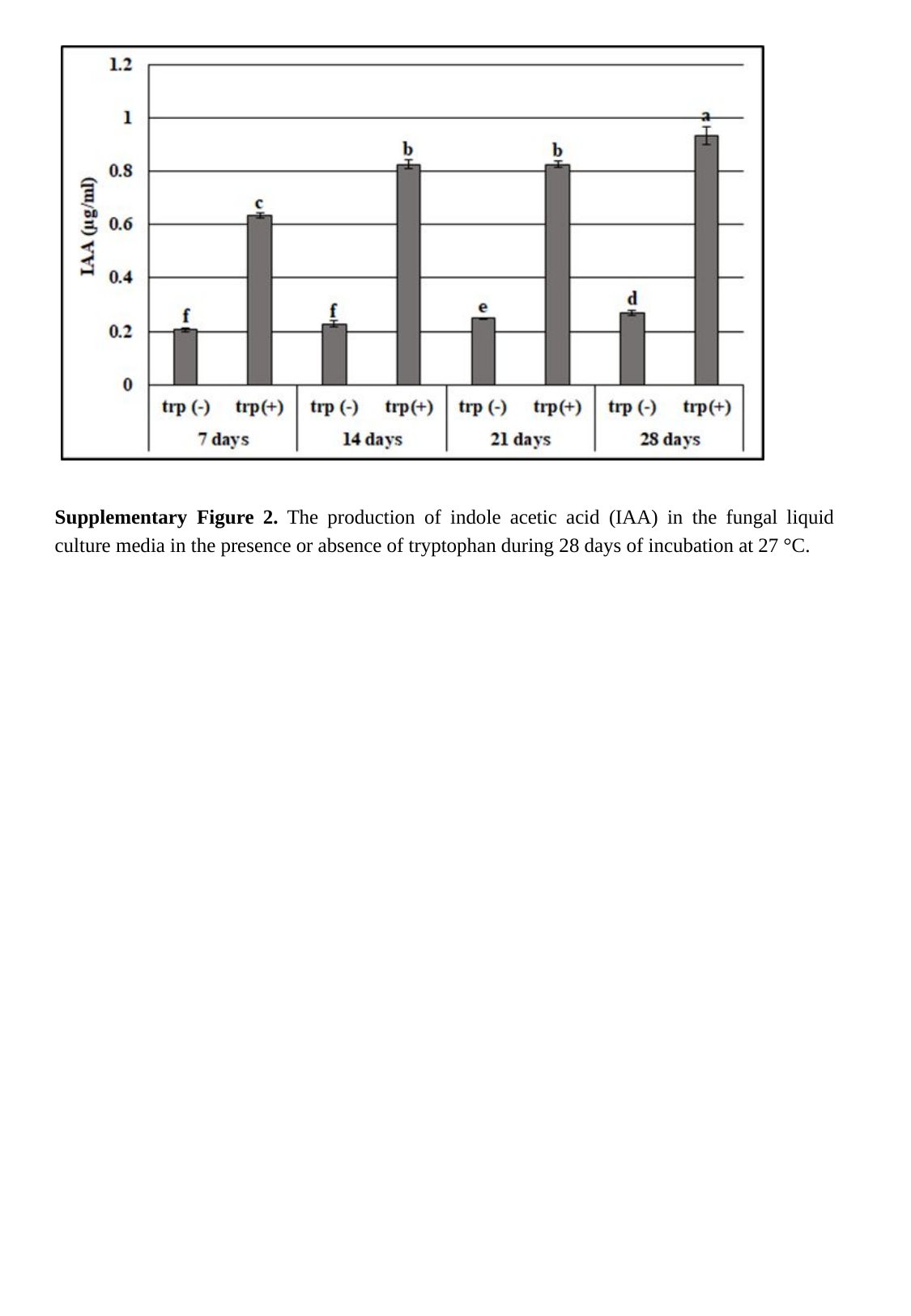

Supplementary Figure 2. The production of indole acetic acid (IAA) in the fungal liquid culture media in the presence or absence of tryptophan during 28 days of incubation at 27 °C.

## Slide 3
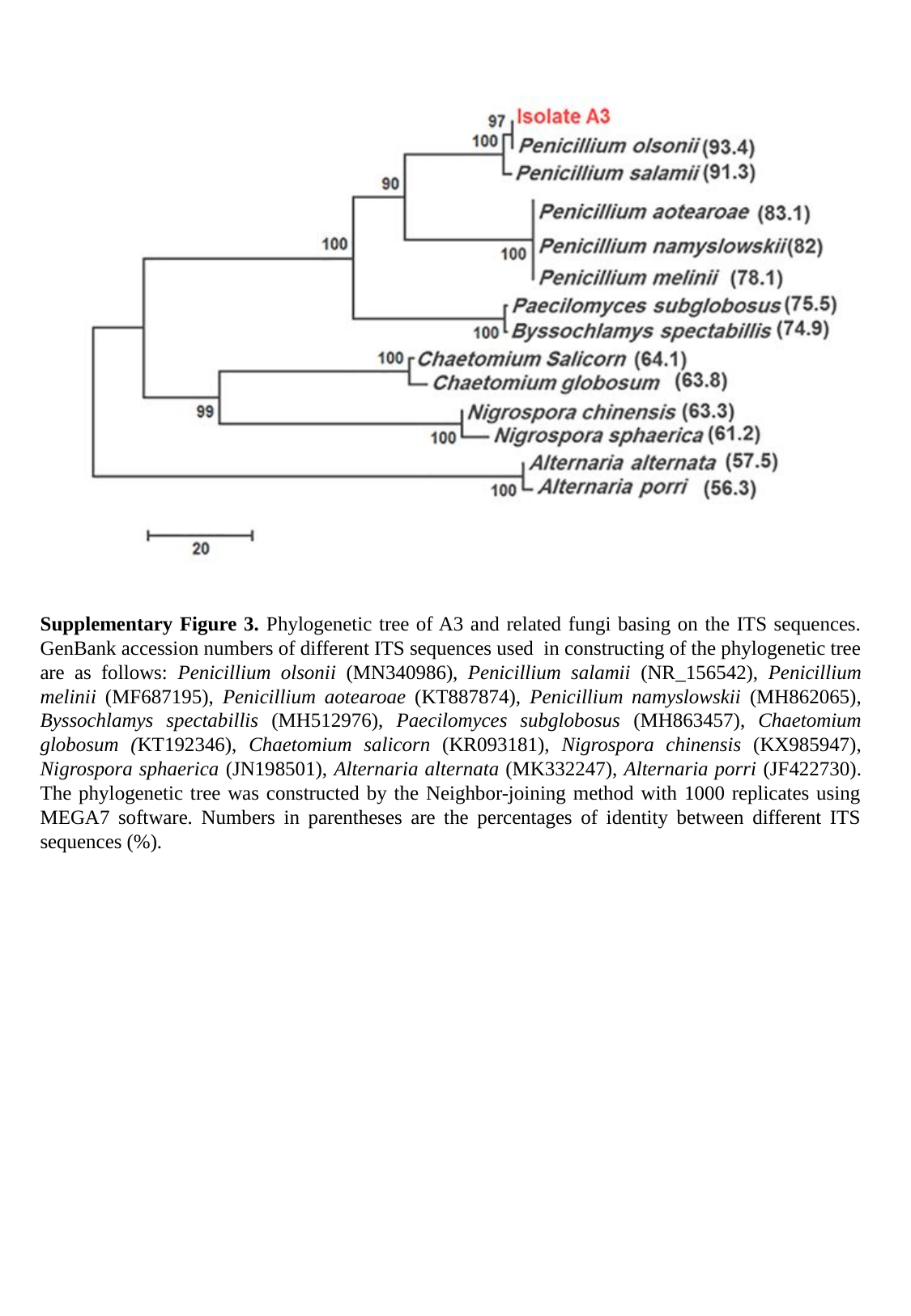

Supplementary Figure 3. Phylogenetic tree of A3 and related fungi basing on the ITS sequences. GenBank accession numbers of different ITS sequences used in constructing of the phylogenetic tree are as follows: Penicillium olsonii (MN340986), Penicillium salamii (NR_156542), Penicillium melinii (MF687195), Penicillium aotearoae (KT887874), Penicillium namyslowskii (MH862065), Byssochlamys spectabillis (MH512976), Paecilomyces subglobosus (MH863457), Chaetomium globosum (KT192346), Chaetomium salicorn (KR093181), Nigrospora chinensis (KX985947), Nigrospora sphaerica (JN198501), Alternaria alternata (MK332247), Alternaria porri (JF422730). The phylogenetic tree was constructed by the Neighbor-joining method with 1000 replicates using MEGA7 software. Numbers in parentheses are the percentages of identity between different ITS sequences (%).

## Slide 4
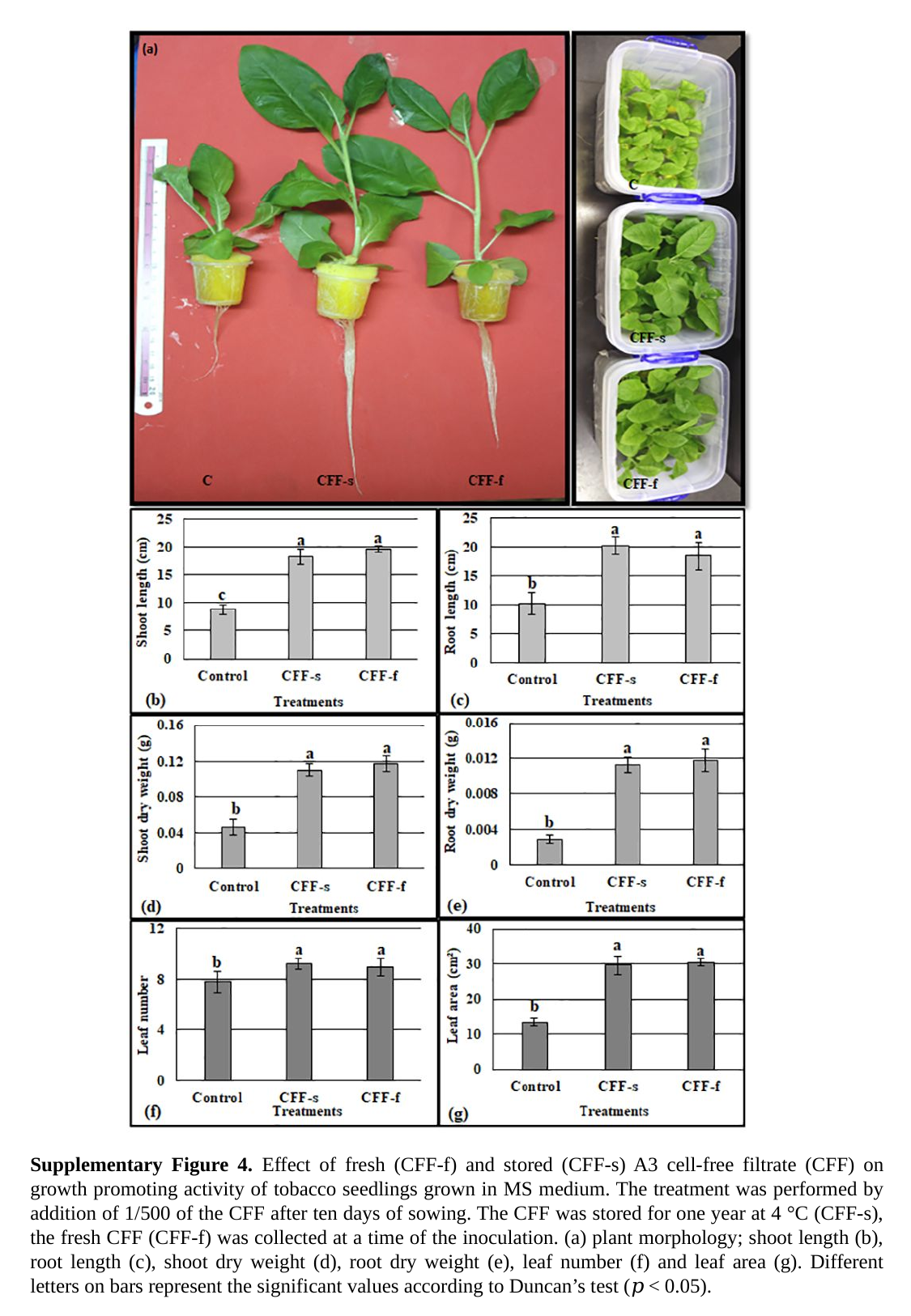

Supplementary Figure 4. Effect of fresh (CFF-f) and stored (CFF-s) A3 cell-free filtrate (CFF) on growth promoting activity of tobacco seedlings grown in MS medium. The treatment was performed by addition of 1/500 of the CFF after ten days of sowing. The CFF was stored for one year at 4 °C (CFF-s), the fresh CFF (CFF-f) was collected at a time of the inoculation. (a) plant morphology; shoot length (b), root length (c), shoot dry weight (d), root dry weight (e), leaf number (f) and leaf area (g). Different letters on bars represent the significant values according to Duncan’s test (𝑝 < 0.05).

## Slide 5
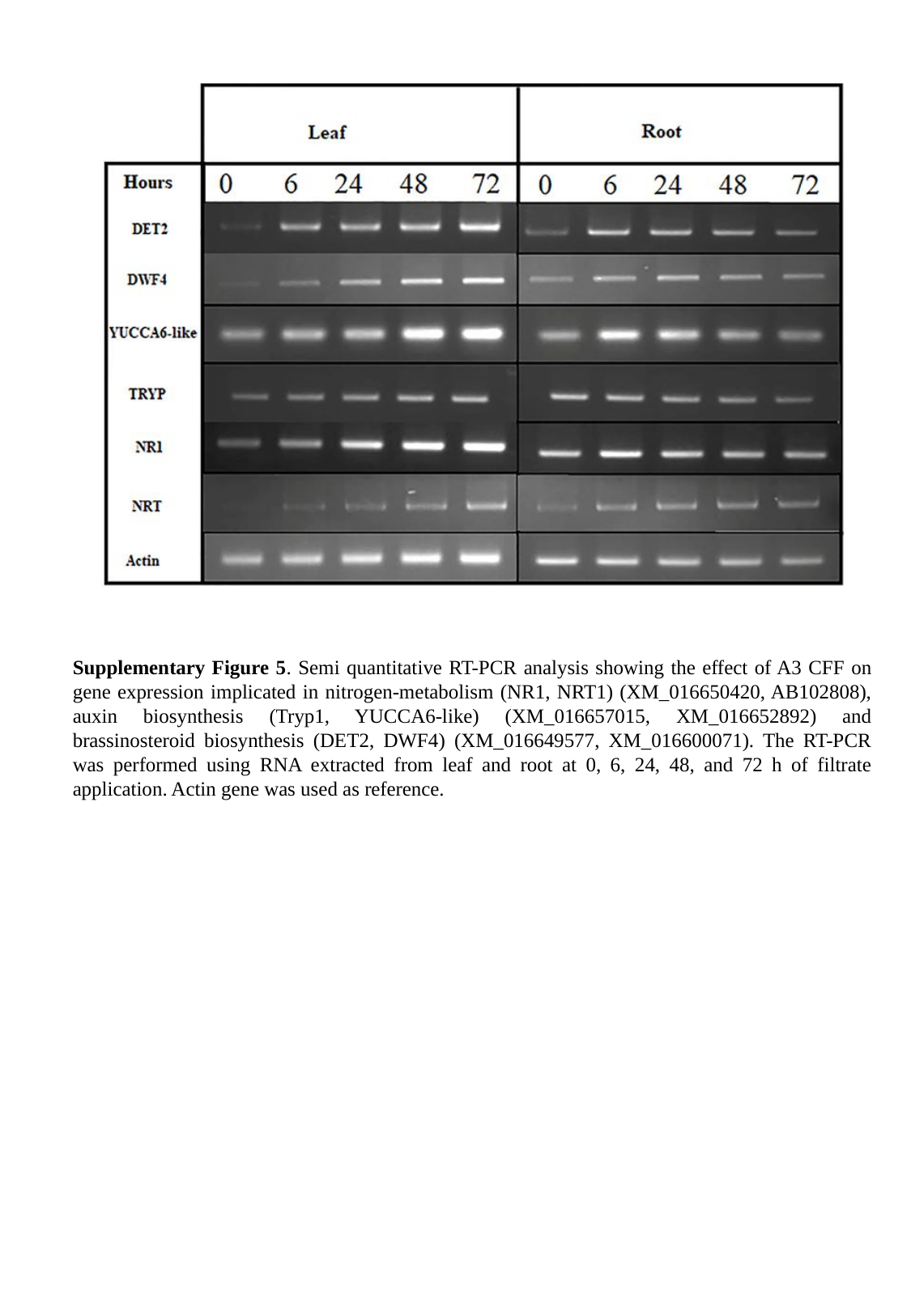

Supplementary Figure 5. Semi quantitative RT-PCR analysis showing the effect of A3 CFF on gene expression implicated in nitrogen‐metabolism (NR1, NRT1) (XM_016650420, AB102808), auxin biosynthesis (Tryp1, YUCCA6-like) (XM_016657015, XM_016652892) and brassinosteroid biosynthesis (DET2, DWF4) (XM_016649577, XM_016600071). The RT-PCR was performed using RNA extracted from leaf and root at 0, 6, 24, 48, and 72 h of filtrate application. Actin gene was used as reference.
