# Supplementary material for: A novel PGPF Penicillium olsonii isolated from the rhizosphere of Aeluropus littoralis promotes plant growth, enhances salt stress tolerance, and reduces chemical fertilizers inputs in hydroponic system
Source: Front Microbiol. 2022 Oct 27;13:996054. doi: 10.3389/fmicb.2022.996054 (PMC9648140; doi:10.3389/fmicb.2022.996054)
Supplement: Supplementary file 2 [file Table_1.DOCX]

| **Primer** | **Sequence (5’--------- 3’)** |
| --- | --- |
| YUC6-F | GGTTTGCTTGGTGCTTCCAT |
| YUC6-R | CTTTTCCTCTTTCACCTTGTCGT |
| Tryp1-F | GCAGAGGCTTATGTCTATTCAAGG |
| Tryp1-R | ATCCAAGTTTCATTGCTGTAAAAAG |
| DET2-F | TTTGGTTCCTAGAGCTTGTGC |
| DET2-R | TCAAGGTAAAGCCAAATACAGTCC |
| DWF4-F | GCCTTTTGCCTTCCCTTACG |
| DWF4-R | CTGAGGTGGAGCCTAACGTC |
| NR1-F | ATGTCACAATACCTTGATTCTCTCC |
| NR1-R | AGTTATCCCTGTTCCACCTGC |
| NRT1-F | AGAGACTTGCTGAAATGGGGA |
| NRT1-R | GATGATGGAGTAACAAGCGCA |
| ACT-F | GTGCCCATTTACGAACGATA |
| ACT-R | GAAGACTCCATGCCGATCAT |
| HKT1-F | CATTGTTCGGTGCTGTCGAC |
| HKT1-R | AATGCCAAGTTCTGCACCAC |
| NHX1-F | TTGATGAGAGGCGCAGTGTC |
| NHX1-R | TTGACTGGCTAGAAGTGGCG |
| CAT1-F | TGCTCCAAAGTGTGCTCATC |
| CAT1-R | GAAGCAAGCTTTTGACCCAG |
| SOS1-F | TATCAGGTGGAGGCTAGAGC |
| SOS1-R | TCATGCTCCCGTACATGCTC |
| SOD-F | AGCTACATGACGCCATTTCC |
| SOD-R | TCCCAAACGTCTATTCCCAG |
| ITS1-F | TCCGTAGGTGAACCTGCG |
| ITS4-R | TCCTCCGCTTATTGATATGC |

**Table 1**. The primers sequences used in used in PCR , RT-PCR and qRT-PCR analysis
